# Smart Electrochemical Portable Tools for Cultural Heritage Analysis: A Review

**DOI:** 10.3390/s19194303

**Published:** 2019-10-04

**Authors:** Federica Valentini

**Affiliations:** Sciences and Chemical Technologies Department, Tor Vergata University, via della Ricerca Scientifica 1, 00133 Rome, Italy; federica.valentini@uniroma2.it

**Keywords:** electrochemical impedance spectroscopy (EIS), metallic corrosion, voltammetric techniques, screen-printed electrodes (SPEs), small sensors, portable immunosensors, biosensors, graphene oxide, cultural heritage

## Abstract

Protecting Cultural Heritage (CH) from corrosion and other environmental damages, mainly involving metallic or organic layers contained in artwork, represents a major challenge for conservation scientists. Electrochemical techniques provide useful information about the deterioration effects of metallic coatings and organic layers. Recently, Electrochemical Impedance Spectroscopy (EIS) has been successfully applied in the study of metallic corrosion. However, EIS has not succeeded in becoming a routine technique, due to problems regarding both instrumental apparatus (which is not ideal for in situ analysis, especially with previous cell configurations), and the difficulty with data processing. At the same time, new portable electrochemical sensors, immunosensors, and biosensors have successfully made a scientific impact, mainly with in situ diagnosis of organic components contained in CH objects. For this purpose, this review presents two sections: the first describes the analytical optimization of impedance electrochemical cell geometries that are suitable for in situ metal-coating investigation; the second reports on the assembly of small electrochemical sensors, immunosensors, and biosensors, which useful for in situ organic layer characterization. This overview summarizes the state of the art regarding the application of electrochemical techniques and small electrochemical devices as alternative tools for the understanding of CH.

## 1. Introduction

**Scheme 1:**
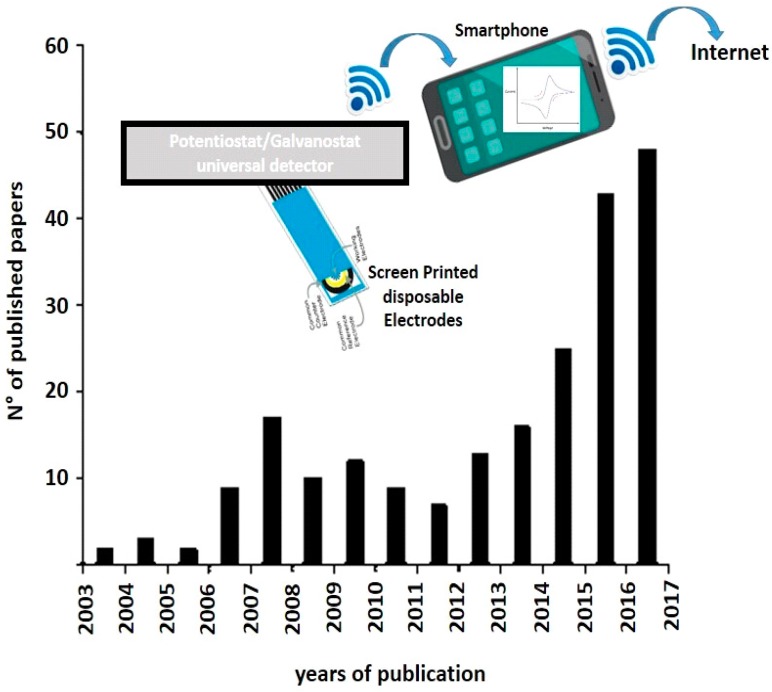
Number of scientific papers per year for smartphone-based chemical sensors, wireless and wearable biosensors, tracked through the Web of Science database, as reported in reference [23]).

Electrochemical sensors represent a specific category of sensors and of miniaturized equipment useful for characterizing the conservation status of Cultural Heritage (CH), both indoor and outdoor artwork objects and surfaces. There are several materials to preserve in CH field applications, ranging from organic matter intrinsically present in the artwork objects and organic protective coating of artwork surfaces, such as waxes, resins, polymers, and composites, to metallic layers and alloys. For example, Raman and Fourier Transformer Infrared Spectroscopy (FTIR) can demonstrate whether an organic layer has serious environmental damage, but not how this change could be involved in redox processes such as the common corrosion, passivation, and etching events. Conversely, X-Ray Fluorescence (XRF) is useful for knowing the chemical composition of metal coatings. Patinas and corrosion secondary products are analyzed by X-Ray Diffraction (XRD), FTIR and Raman spectroscopies, but only for their chemical composition. Moreover, microscopy techniques (such as Scanning Electron Microscopy (SEM)) are suitable for characterizing the morphology of the surface and deep profile of corrosive layers. Electrochemical techniques, especially Electrochemical Impedance Spectroscopy (EIS), provide unique information about the corrosion behavior of metals [1,2,3] and chemical modification during corrosion/passivation events. Other electrochemical tools, such as polarization techniques, provide information on patina stability [4,5,6] without a direct contact with CH surfaces, because polarization is a destructive method (unlike Polarization Resistance (PR), [7,8,9,10,11,12,13]). Although these are useful techniques and complementary to conventional ones, EIS has been delayed in establishing itself in CH fields, mainly due to in situ analysis requirements (assembling small portable devices) and data interpretation because of few general equivalent circuits (necessary for interpreting results). All these aspects are discussed in the first section of this review, and in the second section, portable electrochemical sensors, immunosensors, and biosensors are described for in loco measurements. Portable electrochemical Surface Enhanced Raman Spectroscopy (SERS) [14] is suitable for a highly sensitive and selective diagnosis of organic layers; miniaturized electrochemical cells of the Bragg model [15] are suitable for in situ synchrotron X-ray measurements on heterogeneous CH alloys with corroding surfaces. Screen-Printed Electrodes (SPEs), chemically modified with Graphene Oxide (GO), are extremely highly performing for redox status identification of pigments and earth present in ancient leather samples [16]. Paraloid B72 film-modified microelectrodes are applied for detection of inorganic pigments in paintings, by using cyclic voltammetry and differential-pulse-stripping voltammetry [17]. Regarding portable electrochemical immunosensors, nano-electrode ensembles (NEEs) are fabricated for the recognition of organic binder in paintings [18]. The immunochemical localization of ovalbumin in paint cross-sections can be carried out using high-performing Scanning Electro-Chemical Microscopy (SECM) [19,20]. For electrochemical biosensors, there are two interesting strategies to apply in situ for CH diagnosis: (i) the impedance biosensors to quantify pathogenic bacteria, involved in biofilm assembly [21]; and (ii) Surface Plasmon Resonance (SPR) combined with a biosensor platform to recognize albumen, yolk, or their mixtures in water-based Highly Viscous Polymeric Dispersions (HVPD) extracts [22]. This review is a useful updated overview, which focuses on electrochemical devices such as miniaturizable tools (with a tendency towards wireless transmission of the analytical signal) suitable for field measurements and with great interest from the scientific community, given the number of publications per year, as shown in Scheme 1.

## 2. Electrochemical Impedance Spectroscopy in CH 

### EIS for Evaluating Corrosion Events and Analyzing Metallic Protective Layers of CH Objects and Surfaces

**Figure 1:**
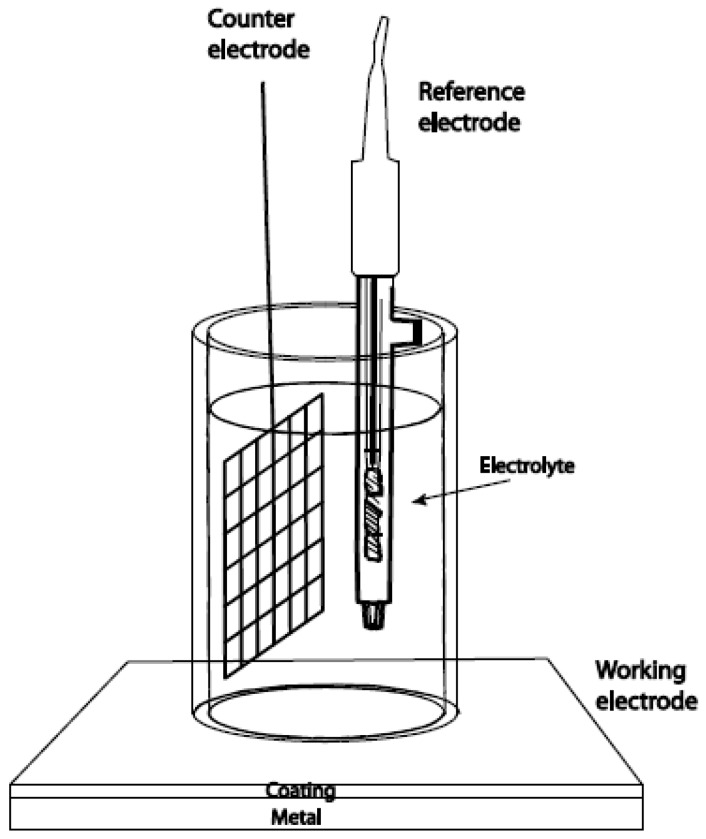
Electrochemical cell for metal detection in CH objects, reproduced with permission from [25]).

The general applicability of electrochemical techniques such as EIS is related to low-conductivity electrolytes. Low conductivity values are required to make the cell impedance electrical contribution non-negligible [24] during measurements. Moreover, the electrochemical cell needs to have an appropriate geometry (Figure 1) to minimize interference with metal surfaces belonging to the CH.

**Figure 2:**
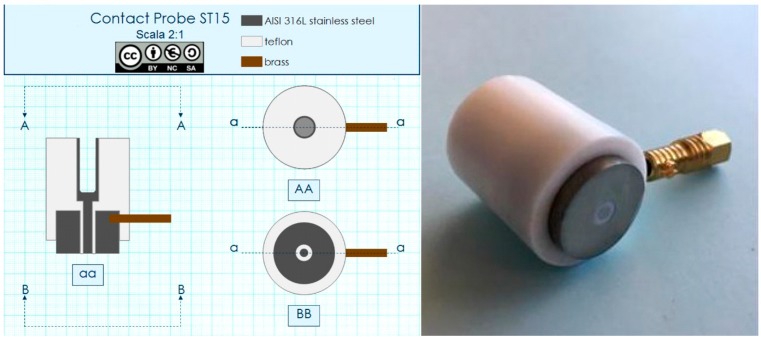
A Contact Probe (CP), reproduced and reprinted with permission from [30].

For this purpose, distances between three electrodes are reduced, to minimize the uncompensated electrolyte resistance. Conversely, the reduced distances can produce capacitive couplings, causing spurious contributions in electrochemical impedance spectra. To solve these problems, several authors developed two in situ electrochemical cell setups. Setup 1 consists of a contact probe-electrochemical cell (the first innovative solution designed by Paola Letardi [26,27]), containing the liquid electrolyte [28,29] that allows a direct measurement of metal coatings. This probe consists of two electrodes: the auxiliary electrode and the secondary electrode (Figure 2).

Setup 2 is a gel cell, with silver/silver chloride secondary electrode and agar or agarose as gelling agent [31,32] useful for immobilizing alkaline chloride solutions, simulating what happens inside the pores of concrete and plaster surfaces [33].

In Setup 1, the probe is assembled into a PTFE (PolyTetraFluoroEthylene) cylinder, combined with auxiliary and secondary reference electrodes (in a typical three-electrode cell setup). Mineral water represents the working electrolyte, directly in contact with the CH surfaces [3,34]. Then, a combination of the electrochemical cell with portable impedance spectroscopy apparatus resembles a unique device, with electronics and data acquisition systems [35,36]. EIS spectra, recorded with the portable/miniaturizable contact probe, provides electrochemical signals that are completely superimposable on those obtained when working with the standard reference cells applied in laboratories [3]. This means that the new proposed contact probe is an analytically validated system, with mineral water or low concentration (typically 0.1 M) of NaCl, as working electrolyte, according to Letardi, Cano et al., [37,38,39,40].

The validated contact probe is directly applied on an outdoor surface, such as the nineteenth statue of Napoleon located in Milan, to evaluate the efficiency of the restoration treatments [29,41,42,43]. In situ experimental results were comparable to those observed in the laboratory from bronze coupon samples [3,26,39,40,41]. In particular, |Z| is the measured parameter that resulted in a 10 mHz value, after in situ experiments, meaning that different areas showed different values of corrosion susceptibility. This is a compatible and reasonable result, considering that different areas of the metal surface could have suffered different damage, presenting different values of corrosion susceptibility. Furthermore, the contact probe is suitable not only for the diagnosis of environmental damage but also for evaluating the efficiency of the restoration treatment, establishing its non-invasive performance with respect to the treated metal surfaces. In particular, the selected statue of Napoleon was cleaned using the laser technique, and Letardi, Cano et al. [29,41,42,43] demonstrated that after the laser treatment, a |Z| value recorded on the cleaned bronze sculpture was lower, but higher than that detected on the original cleaned bronze surface (the control sample, used here for comparison). This experimental result demonstrates that the laser cleaning protocol is suitable for a non-invasive metallic surface restoration (belonging to historical surfaces and CH objects), avoiding the etching/corrosion/passivation phenomena of the original bronze matter. Unfortunately, the contact probe suffers from experimental limits, such as its rigid flat cell geometry being inappropriate for outdoor monument measurements. To solve this problem, the authors have developed solid electrodes combined with flexible cells, described in Setup 2. Another critical point exhibited by the contact probe, and in general by the EIS techniques, consists of the difficulty of processing the acquired experimental data. Most authors only consider the |Z| parameter, recorded at low frequencies, where its corresponding values range from 10 to 50 mHz, mimicking the two extreme values, where for low impedance values, the metallic corrosion occurs easily, while for high-impedance values, corrosion occurs much more slowly, i.e., the phenomenon of damage is slowed down. The same authors [44,45,46] have also monitored the |Z| evolution profile with immersion time and acceleration tests. In both approaches, the Nyquist equivalent circuit (EC) is applied for data interpretation, but the values of the different components are not reported, and neither the discussion of the applied model nor the curve fitting are described in the literature [47,48]. These listed critical points make the EIS technique, coupled with the contact probe, not yet mature enough to be used as a routine technique in the field of analytical diagnosis for the conservation of metallic CH coating, layers, and surfaces.

**Figure 3:**
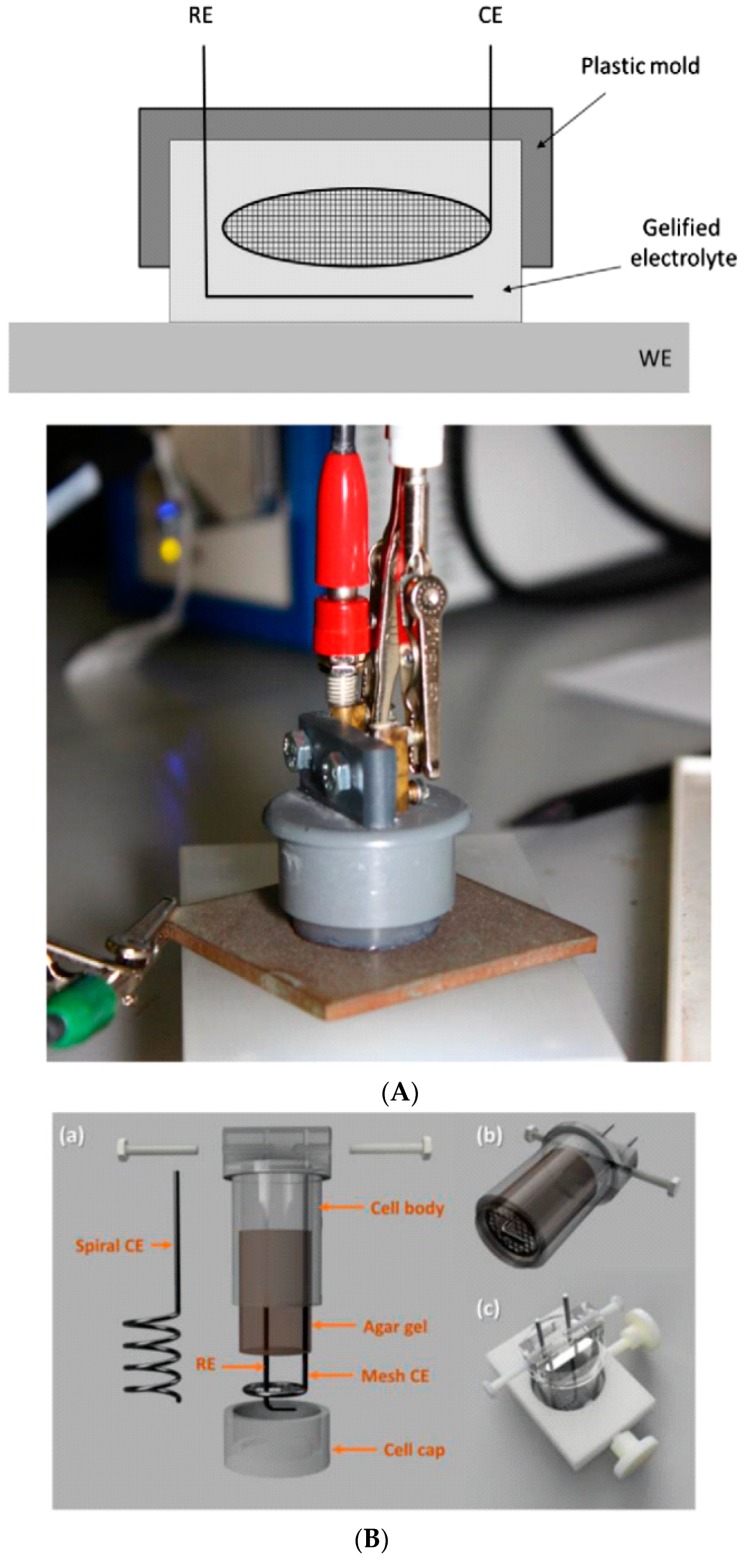
(**A**): Solid agarose gel electrolyte: scheme (up) and photograph (down); (**B**): zoom of a solid agarose gel. Reproduced and reprinted here with permission from [30] and [31], respectively.

The best strategy to improve the experimental limits exhibited by contact probe technology consists of the application of solid electrolytes housed in the previous ECG gel-based electrodes (provided by Angelini, et al.; [46]), in modern hydrogel cells, designed by Clare et al.; [49] and finally in an updated alternative, proposed by Cano et al. in reference [31]. The latter consists of an application of a liquid electrolyte, gelled with agar, in a solid agarose gel electrolyte prototype (labeled G-PE). Low concentration of agar provides similar results to that obtained when working with traditional liquid electrolyte, according to the literature [32]. The new design of the G-PE cell (Figure 3) has been optimized using a new cylinder base to be longer and thinner, with 2 cm diameter, mainly realized with transparent methacrylate [50].

**Figure 4:**
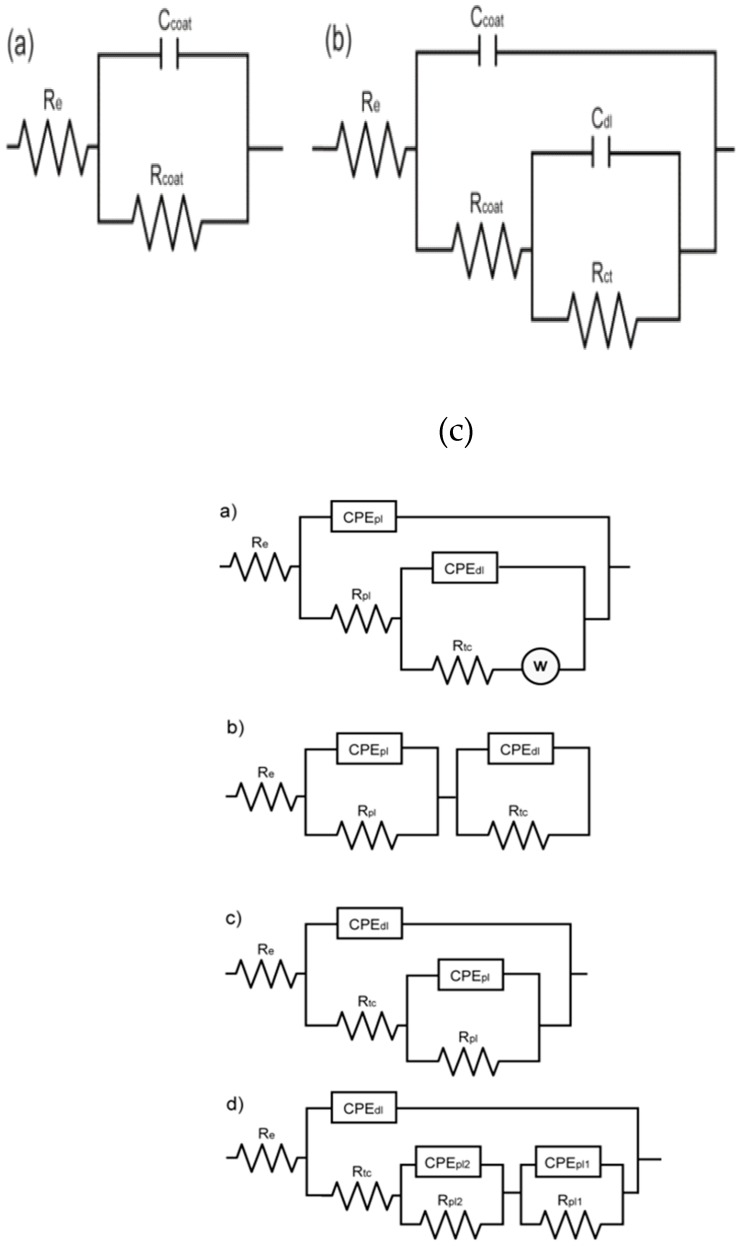
(**a**)**:** EC showing an ideal metal-coating system; (**b**) a damaged coating; and (**c**) different EC schemes proposed to represent archaeological copper alloys. Reproduced and reprinted with permission from [41].

This new geometry, suggested by Cano et al. in reference [31], allows the filling of the cylindrical electrochemical cell with liquid electrolyte, providing a comparative calibration/validation procedure between the usual laboratory setup for EIS measurements and the G-PE cell (which was previously referred to as Setup 2), when the experiments are carried out in situ. In this case, working with low-conductivity electrolytes [50], the geometry of three electrodes minimizes the ohmic drops and current density inhomogeneity (two effects that should be minimized to avoid compromising the signal-to-noise ratio). The electrolytic working medium was prepared by gelling a liquid electrolyte with agar, where the liquid electrolyte is the artificial rain (the latter was selected as it best represents the external environments with which the metal surfaces of CH are in contact). Ramirez Barat and Cano [50] chose the bronze sphinx at the facade of the Spanish National Archaeological Museum (MAN) in Madrid to perform a comparative evaluation (i.e., an analytical validation) between the new portable G-PE-based cell, equipped with the solid electrolytes, and the laboratory-cast bronze coupon samples. The two bronze sphinxes were previously protected with Incralac [51] and the resulting EIS measurements were comparable to data collected in the case of the bronze coupon samples. The |Z| parameter (mainly considered to be low-frequency-range values as a total evaluation of the corrosion resistance [52]) is useful to establish whether a metallic layer is working better than other coatings. In particular, the |Z| parameter is strictly related to the protective properties of the environmental patina that affect the metal coating. In this case, |Z| can be calculated from the diameter of the high/medium-frequency semicircle represented in a typical Nyquist plot [53]. Similarly, the charge transfer resistance (Rct) parameter is also evaluated by the low-frequency semicircle [54] in the Nyquist plot. The main problem with this strategy concerns the difficulty in defining in a precise and accurate manner the semicircle in the Nyquist plot profiles (as already mentioned above in the case of data processing acquired with the contact probe technique) that usually appears to be quite overlapped, leading to wrong estimations [55] of the original corrosion phenomena. The EC houses passive electric elements, such as resistors (R) and capacitors (C) that reproduce the electric features of the original metallic layer very well (Figure 4). Unfortunately, the metal surface that covers a lot of artwork objects is heterogeneous, inhomogeneous, and very porous, with several irregularities [56,57]. In this case of study, an EC applies a constant phase parameter (i.e., CPE_Constant Phase Element) instead of pure capacitors, with an impedance parameter equal to Z_CPE_ = 1/Y_0_(jω)^α^. Instead, when in the presence of a capacitor, the global interfacial impedance parameter is equal to Z_e_ = Z − 1/(jωC), where Z_e_ is the global ohmic impedance and C is pure capacitance in units of F/cm^2^. Working and recording the analytical signals at the high frequency range, Ze tends towards Re, which corresponds to 1/jωC value, so finally Z_e_ is equal to 1/jωC. When using CPE, it is important to underline that a CPE simulates the behavior of a capacitor and the Y_0_ value coinciding with the capacitance only for α = 1 value. In general, for α < 1, it is advisable not to consider Y_0_ as an equivalent of the capacitance parameter, incorrectly using the same units of measure, such as F (s/Ω). The correct Y_0_ units are S·s^α^ or s^α^/Ω, and thus Y_0_ is not a capacitance, especially in the presence of real/original CH samples with metal covers that appear completely inhomogeneous [58,59].

According to these considerations, the first approach for a correct evaluation of EIS experimental data, collected during measurements of CH metallic coatings/layers, is to correlate different electrochemical responses/profiles with different real cases of studies, as summarized on Table 1.

**Scheme 2:**
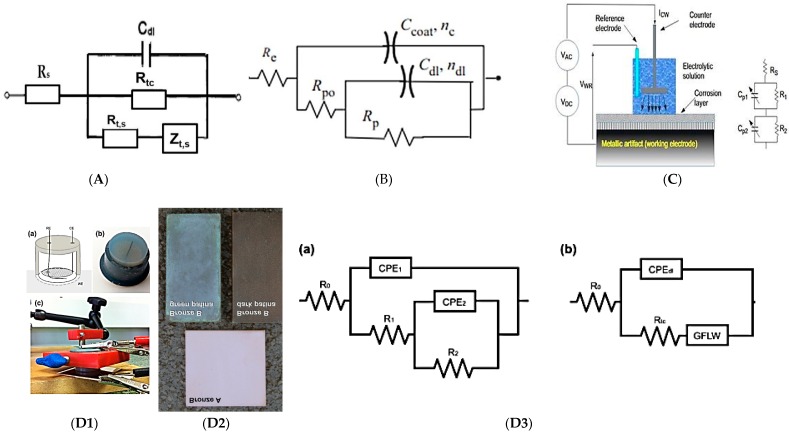
Equivalent Circuits (ECs) reported in Table 1, in particular: (**A**) represents the AC: Alternative Current Impedance Spectroscopy for copper in simulated tap water. (**B**) Represents the electrical equivalent circuit used for fitting EIS data in the presence of copper/alloy-coated patina. (**C**) Shows the EIS measuring principle and the corresponding EC carried out on iron/steel-coated patina. (**D1**) Cell design scheme (on the left); bronze coupons used for the electrochemical tests (in the middle **D2**) and the Equivalent electrical circuits (**D3**) used to analyze EIS data: (**a**) is the equivalent circuit with 2 nested CPE-R pairs, and (**b**) is the second CPE replaced by a generalized finite-length Warburg impedance.

Among the most representative case of the original metal samples, reported in Table 1, the EC described in the second column has been also shown using the corresponding equivalent circuits, all organized in Scheme 2.

At the end of the first section, in Table 2, a summary of the best analytical performance of Setup 1 and Setup 2 is reported to highlight the optimized experimental conditions to use EIS-based sensors for metal corrosion investigation, directly applied on artworks and their surfaces.

## 3. Electrochemical Devices to Apply in CH 

### 3.1. Electrochemical Small Tools to Apply in Diagnosis and Preservation of CH

#### Portable Electrochemical Sensor Prototypes in CH Field Applications

**Figure 5:**
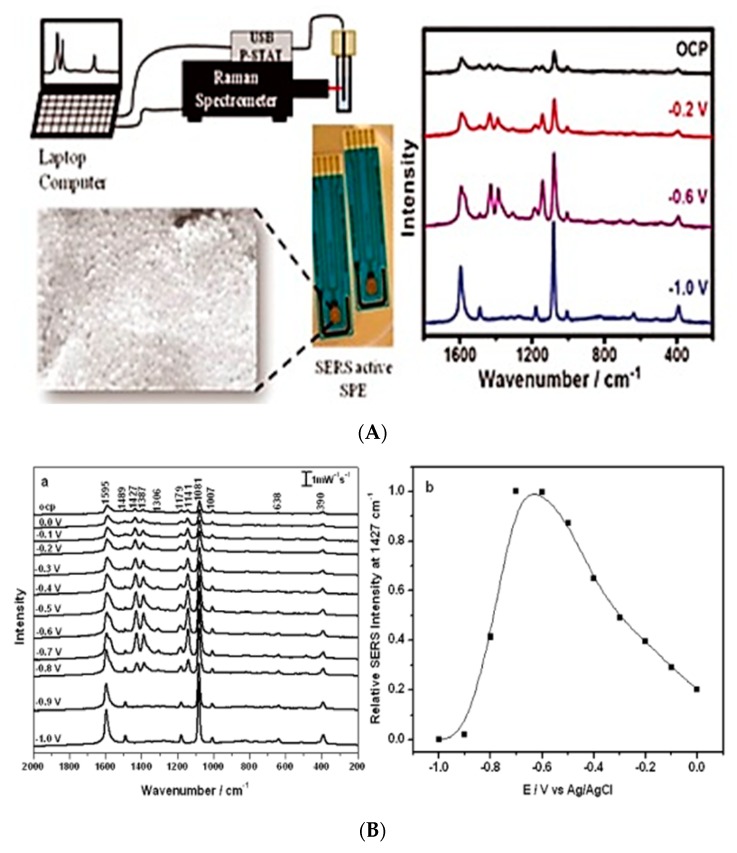
(**A**): Scheme of the miniaturized equipment with a SEM micrograph of the electrode surface, with a typical Raman spectral profile, as output signal. (**B**) Shows on (**a**) a typical electrochemical Surface Enhanced Raman Spectroscopy (SERS) profile for a para-aminothiophenol modified electrode; (**b**) a typical SERS profile (for a specific analyte, such as p,p′-dimercaptoazobenzene_DMAB) depending on its redox potential. Reproduced and reprinted with permission from [14].

In this section, portable electrochemical devices are described with their specific applications particular to the analysis of organic pigments and inorganic and composite-based materials that comprise artwork objects. As a first prototype of the second section of this review, the bench-top Raman spectrometer, equipped with potentiostatic apparatus [14], is reported, together with its main applications. This smart apparatus can perform Raman analysis and cyclic voltammetry measurements, simultaneously. This is a considerable advantage compared to apparatus that only make Raman measurements, because even coupling the electrochemical information succeeds in offering a complete analysis, especially in the presence of ancient organic pigments (for which it is important to know the state of oxidation). The electrochemical cell is a standard glass vial with a working electrode and a reference electrode, with a special adapter to connect with the potenstiostat Raman spectrometer (Figure 5).

In this specific application, the Surface Enhanced Raman Spectroscopy (SERS) active screen-printed electrode system was prepared by drop-casting of 5 microliters of Ag nanoparticles [14]. Dispersion of nanoparticles was fabricated using the standard Lee and Meisel preparation method, according to the literature [78]. Briefly, the citrate-reduced silver colloids and the reduced nanoparticles exhibited a peak absorption wavelength of around 420 nm, combined with a Full Width at Half Maximum (FWHM) of ∼100 nm. The calibration test of the portable electrochemical SERS apparatus was carried out using two analytes: p-aminothiophenol (pATP) and melamine. Both electroactive targets have been investigated in the wavenumber range values of 200–2000 cm^−1^. The spectroelectrochemical profile of pATP is very well known because the probe is widely employed for the calibration of SERS portable equipment. For the melamine, this study [14] represented the first electrochemical SERS application investigating the detection of the melamine electroactive probe, carried out in milk (as a real matrix application). Melamine has a high density of nitrogen atoms in a heterocyclic compound, and is important as a raw material for making polymers. Melamine as formaldehyde represents the raw material for the preparation of melamine resins and/or thermosetting resins, widely used in CH field applications as organic protective layers (for the preservation and conservation of CHs). Until now, organic materials widely applied in CH fields have been detected only using SERS, according to Brosseau, et al. [79]. These authors described the SERS technique directly applied to red pigment belonging to an important historical watercolor by Winslow Homer (1836–1910). These authors only reported a qualitative SERS analysis, suitable for identification of colorants, biomaterials as dyed, natural textiles, linseed oil biofilms present in oil paintings, etc., but did not provide a determination of organic molecules present in CHs. This experimental limit could be overcome by combining electrochemical techniques with SERS-Raman spectroscopy. This is the case of melamine detection, carried out by Robinsons, et al. in reference [14], where applying the electrochemical SERS technique, a very low detection of limit of 5 ppm (mg/L) for melamine was successfully reached. The lowest Limit Of Detection (LOD) and the highest sensitivity towards several organic compounds, widely used in CHs, could offer useful information to conservator scientists and restorers, especially when they need to select the best restoration/preservation practices (especially when there are traces or ultra-traces of organic pigments to be measured and identified). Furthermore, low cost measurement and rapid analysis to make this electrochemical SERS technique applicable for other analytical research fields.

**Figure 6:**
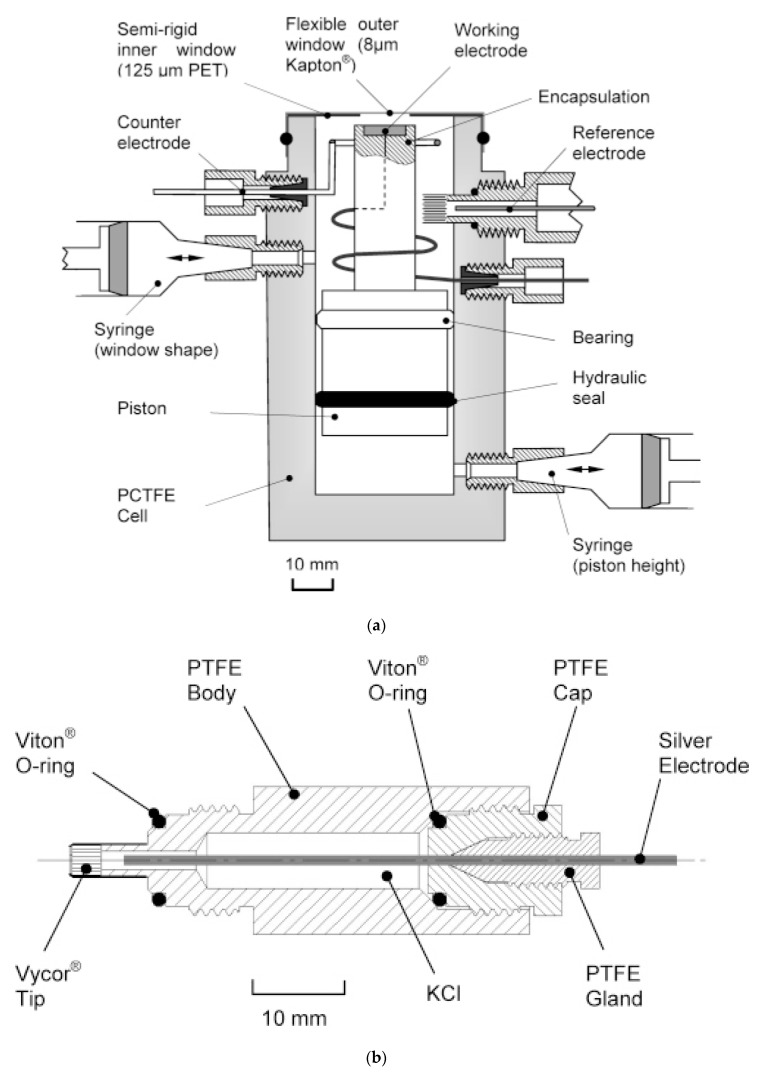

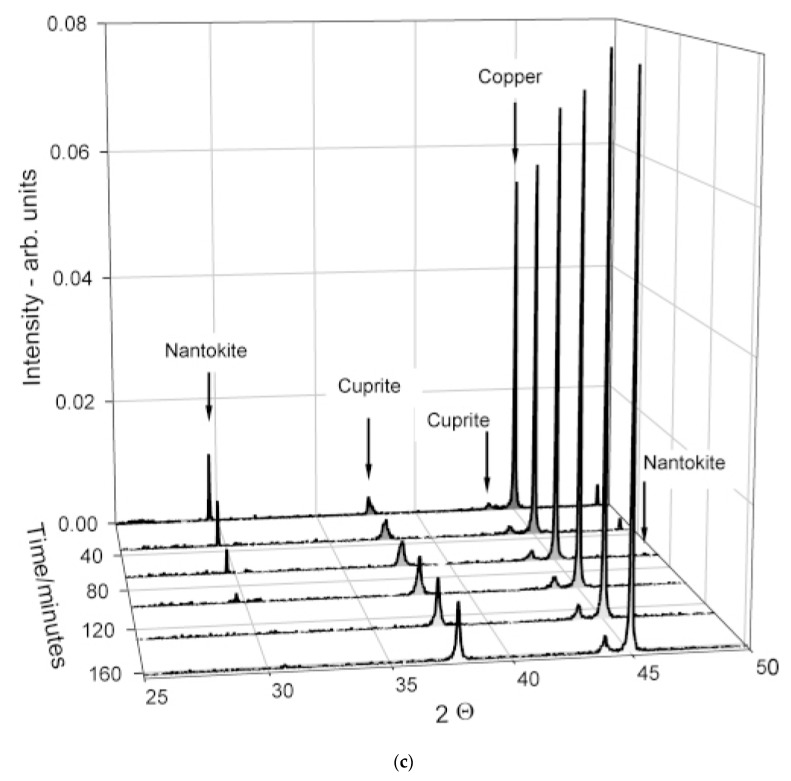
(**a**): Scheme of the experimental setup for a electrochemical impedance measurements combined with a simultaneous X-Ray Diffraction (XRD) analysis of the resulting secondary corrosion metal compounds produced during the deterioration events that occur on metallic coating, present on Cultural Heritage (CH) surfaces; (**b**): Cross-section of the reference electrode. (**c**): The time-resolved spectra typical profiles. Reproduced and reprinted with permission from [15].

The second electrochemical small-sensor prototype consists of an electrochemical cell, working also as a Bragg tool [15], suitable for in situ synchrotron X-ray measurements (Figure 6). The cell is typically of the Bragg model and it represents working electrodes (with a diameter of 16 mm, for the electroactive area). The final dimensions are external diameter of 60 mm and height of 100 mm.

For this experimental setup there are two engineering problems: (1) the cell contained a corrosive solution that potentially causes serious damage to the apparatus; (2) to promote efficient electron-transfer processes, a low-impedance path of the working electrode is required. To solve the first problem, a closed electrochemical cell was assembled, and the X-rays enter through a thin polymer membrane [15]. In this way, the optical windows are mechanically reliable, and the X-ray source provides 3 × 10^14^ photons cm^−2^ s^−1^, with an accelerating voltage of 9 keV, for several days. To overcome the second problem, the working electrode is located at the end of a piston that is hydraulically moved by electrolytes. To carry out X-Ray Diffraction measurements, the working electrode is constantly in contact with the inner window. The geometry of the aperture (a very narrow window with small dimensions) ensures that the spontaneous diffusion process is guaranteed to be the maximum level of analytical performance, providing the appropriate amount of metal corrosion product on the surface of the working electrode, which is required for successfully completing an XRD measurement. An additional control of the electrolyte thickness is reached by setting a small negative or positive hydrostatic pressure in the top of the cell, using the syringe above the piston. Retracting the piston moves the working electrode to an “electrochemical position” able to provide the right electron-transfer processes (avoiding the limitations related to the small volume of the fluid in the pocket). Therefore, the presence of upper and lower syringes guarantees to inject and to remove the same amount of the electrolytes (from above and below the piston), ensuring that the total electrochemical cell volume remains constant for the entire duration of the measurements. Working with this optimized cell geometry, experiments were performed using a monochromatic beam, with a wavelength of 1.6 Å. Slits were positioned so that an area of 2 mm^2^ was explored. Typically, the detector scanned the range 24–50° with a step size of 0.02° (counting time 1 s/step). The photon intensity was in the range 109–1010/s, and each spectrum was collected in the time range of 20–30 min. Electrochemical data were simultaneously collected using CHI1232 handheld potentiostatic apparatus, where an Ag/AgCl/Cl^−^ electrode was applied as a reference secondary electrode. The optimized electrochemical cell configuration was previously tested on artificial samples (before measuring the original and damaged metallic CHs). Laboratory samples, previously stored in a sodium sesquicarbonate solution, were prepared by electrochemical etching of their metallic coatings, thus inducing the formation of the corrosion patina.

Archaeological copper objects extracted from marine environments often have a covering of natural patina, highly appreciated for its aesthetic value and protection towards the underlying metal support. For this purpose, it is called patina nobile, which needs to be preserved and studied. This patina is chemically unstable, especially when it contains chloride/Cl^−^ anionic species. Indeed, the underlying metal corrodes very quickly in the presence of an oxygen-rich atmosphere. Therefore, it is sensible to store these objects in a solution until some diagnostic analysis and/or preservation treatments can be carried out. Sodium sesquicarbonate solution (mentioned above) remains the most common storage procedure, because it is the least invasive towards the patina (compared to all other known restoration treatments [80,81]). The authors [15] monitored the corrosion potential parameter, E_corr_ that results in a constant and stable value, when no modification on the surface composition affected artwork objects. In particular, the same authors [15] also applied a time-resolved in situ Synchrotron X-Ray Diffraction (SR-XRD) technique to verify the corrosion profile of the copper-based alloys. For example, in this work [15], nantokite (i.e., CuCl) represented the electroactive probe/target, able to retain and preserve its main electrocatalytic activity during the entire occurrence of the metal corrosion events. Experimental results show that the combination of X-ray data (able to detect different crystallographic phases that could coexist, when serious corrosion events occur) with the electrochemical data (such as the corrosion potential E_corr_ values), unequivocally provide important information about the nature of metallic corrosion phenomena, suggesting useful restoration/preservation strategies that could be adopted. Finally, the proposed analytical method [15] results are very easy to handle, especially for in situ analysis in museum, libraries, and archives.

**Figure 7:**
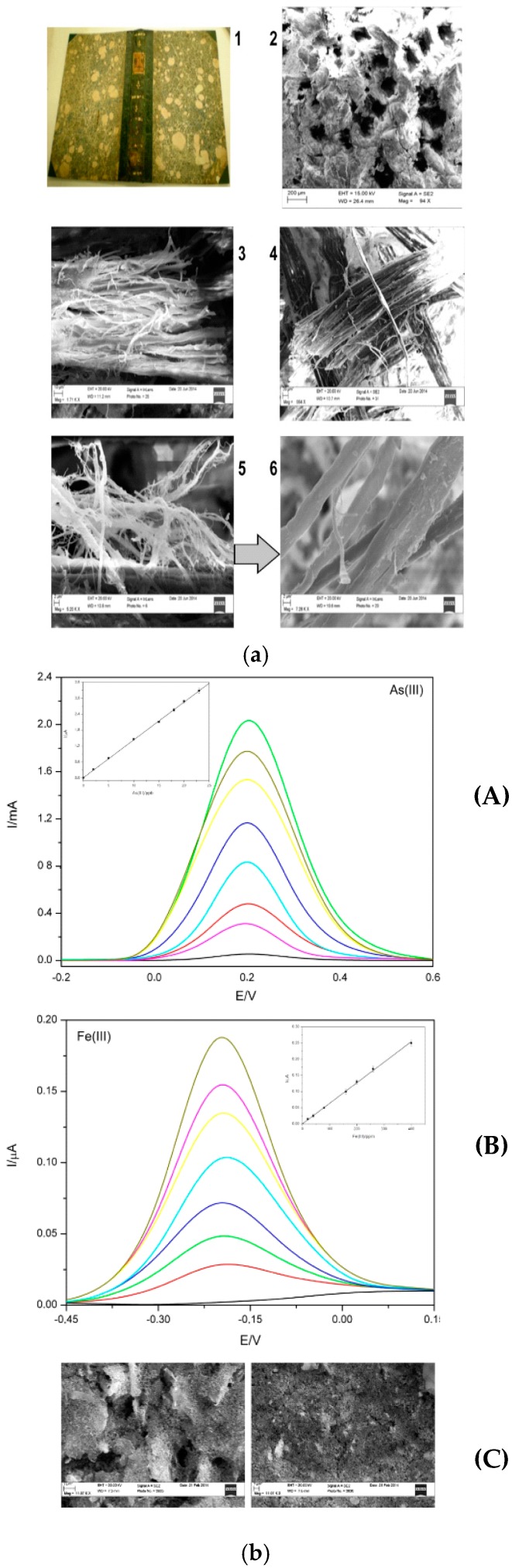
(**a**): **1** is the image of the original leather cover. From **2** to **6**: SEM of the leather samples (flower side and flesh side of the skin/leather samples). In (**b**): (**A**) represents the calibration for As(III), (**B)** represents the calibration for Fe(III), (**C**) shows FE-SEM/EDAX micrographs, before (left image) and after (right image) GO modification of SPEs. Reproduced and reprinted with permission from [16].

The third prototype of the review is represented by SPEs chemically modified with GO, for the identification of the pigments and earth present in ancient leather samples [16]. SPEs modified with GO were assembled by Valentini, et al.; in reference [16], where GO was synthesized by the unzipping reaction of Single Wall Carbon Nanotubes (SWCNT, used here as precursor), according to reference [16]. SPEs were chemically modified by drop-casting, using 5 µL of 1 mg/mL GO-ethanol dispersion, and leaving the solvent to evaporate at room temperature. The as-assembled GO/SPEs electrochemical sensors were applied for the simultaneous and selective detection of As(III) and Fe(III), providing great analytical advantages of a unambiguous attribution about the chemical composition of the green pigment, in a deteriorated leather cover of an ancient manuscript (leather samples belonging to the Bibliotheca Nazionale, Roma). The high analytical performance of the GO/SPEs have certainly improved the information produced by all the other techniques applied in this study: Micro-Raman spectroscopy and X-Ray Fluorescence techniques, especially in terms of the redox status of the elements contained in the pigments (strictly related to the conservation status of them). The experimental results obtained in this work demonstrate the presence of As(III), probably As_2_S_3_ and Fe(III), characterizing the red earths, which are both responsible for the green pigment in the leather sample, Figure 7. The quantitative data, collected by Square Wave Cathodic Stripping Voltammetry (SWCSV) were also confirmed for Fe and As elements, by Inductively Coupled Plasma-Mass Spectrometry (ICP-MS). Voltammetric techniques, applied for the identification of the redox status of the elements, contained in pigments, are useful for understanding two important aspects: the chemical/physical composition of the colors, and the oxidation status and conditions of the elements, useful for selecting the best restoration strategies. In particular, the SWCSVs provide quantitative analysis with excellent reproducibility, when compared with standard analytical techniques widely applied in quantitative analysis, such as ICP-MS (see Table 3).

The fourth prototype of the second section of this review is the micro-sample coatings in Paraloid B72 film-modified electrodes, able to record inorganic pigments in paintings and polychromed sculptures, by using cyclic voltammetry and differential-pulse-stripping voltammetry [17]. Micro-samples were collected from polychromed sculptures, wall paintings, canvas paintings, panel paintings, and altarpieces from Spain, Ethiopia, and Italy from the twelfth to the twentieth centuries. The pigment-coating electrodes were prepared according to the literature [82]. Briefly, 0.1 mg of the pigment was dispersed in 0.1 mL of 0.5% acetone solution of Paraloid B72 acrylic polymer, and the chemically modified electrode was prepared by drop-casting a few microliters of the prepared dispersion onto the surface of a glassy carbon bare electrode. In the first case study, mainly cadmium, copper, and lead, as well as many other transition metals, could be simultaneously detected and co-plated, provoking secondary intermetallic compound generation, with a passivation of the electrode surfaces (electrode fouling effects). In the presence of the passivation events on the electrode surfaces, more selectively modified devices are required [83,84]. Alternatively, according to the literature [17], some components can exist as separate micro-crystallites in the original sample, minimizing the co-plating interferences. Finally, in this study [17], results obtained in the analysis of pictorial artwork samples by applying differential-pulse voltammetry agree well with those obtained by working with traditional Polarized Light Microscopy (PLM), SEM/EDX, XRD, and FT-IR (Fourier Transformer-Infrared Spectroscopy) techniques, on the original CH objects, respectively. Application of chemically modified electrodes to pictorial samples offers a great advantage because they require only a few micrograms of sample materials (the typical amount required in CH fields, ranging from nanogram-to-microgram levels), combined with a low LOD. Several disadvantages, such as fouling effects (i.e., electrochemical passivation phenomena) due to the organic compounds that spontaneously adsorb on the electrode surfaces, and the matrix effect provoked by inorganic binding media and varnishes that can produce interferences toward the electrochemical response of the specific antigens, could occur.

At the end of this paragraph of the second section of the review, it seems to be reasonable to summarize in a table (Table 4) all the main performances (combined with advantages and disadvantages) of the all presented electrochemical sensors dedicated to CH study.

### 3.2. Portable Electrochemical Immunosensors to Apply in CH Fields

**Figure 8:**
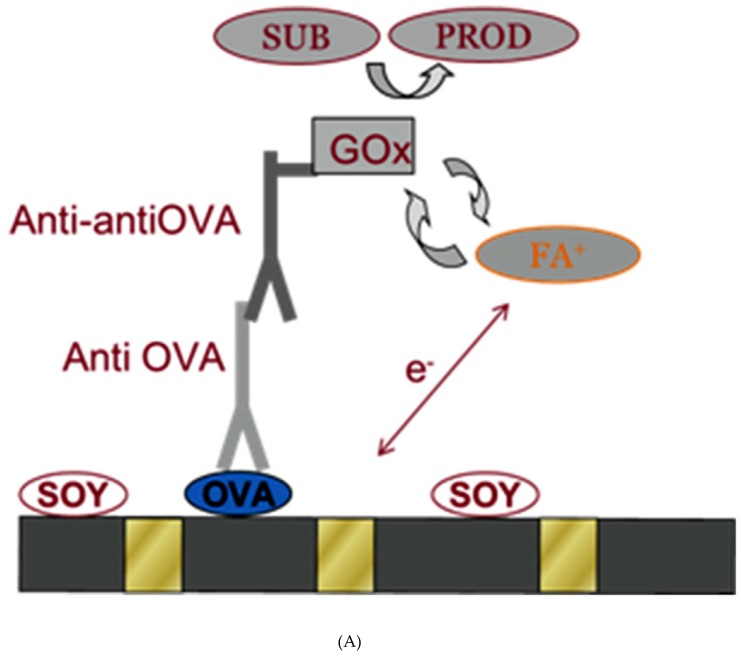

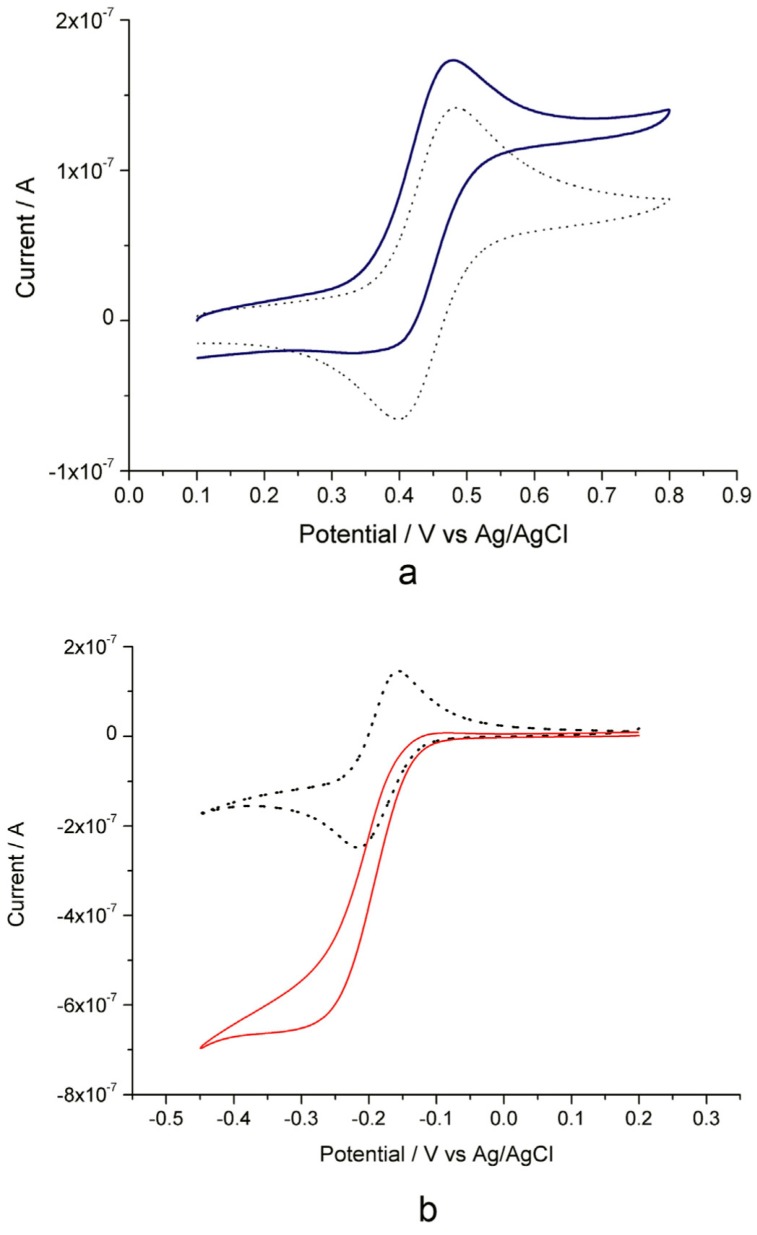

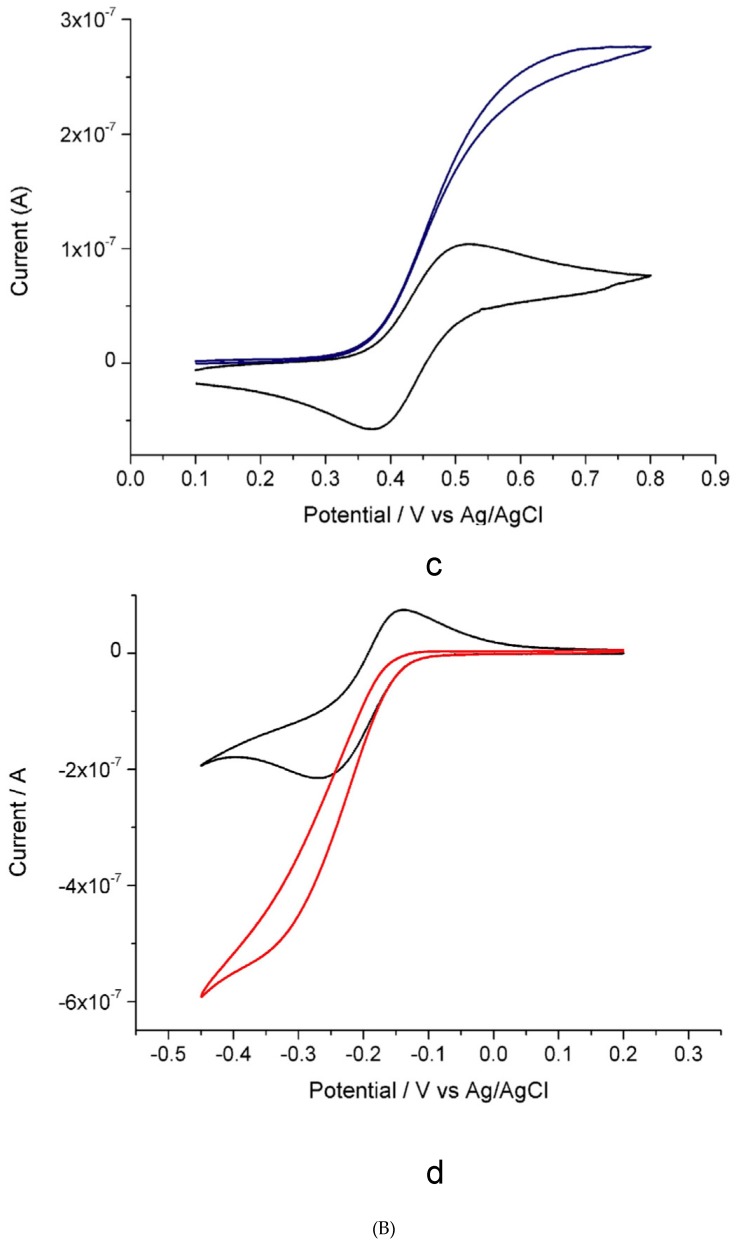
(**A**): Scheme of the immunosensor. (**B**): Cyclic Voltammograms reported on the left show: (**a**) CVs of OVA-immunosensor after incubation in the extract canvas, before and after the addition of glucose; (**b**) CVs of methylene blue at NEE IgY immunosensors incubated with canvas sample, before and after the addition of hydrogen peroxide. (**c**) and (**d**) show the same conditions of (**a**) and (**b**) but working with the original sampled Virgin. Reproduced and reprinted with permission from [18].

In this paragraph, there are different electrochemical immunosensor prototypes, highly selective for the specific antigen–antibody complex reactions, to which it is possible to bring back numerous pigments and organic compounds (which behave as antigens to be determined). The well-known enzyme-linked immunosorbent assays (ELISA) test has been widely applied for protein identification in artwork pigments; however, the immunochemical imaging methods/techniques provide great opportunities to localize the target analytes in wall painting layers, increasing the quality of information and reducing the number of measurements. In this section of the review, there are two cases of study. The first one concerns NEE immunosensors for the detection of ovalbumin, used as a binder in tempera paintings [18]. The second one concerns egg yolk detection, recording the immunoglobulin IgY on the NEEs. In this field of application, there are several template membranes to assemble NEEs, but the affinity of polycarbonate membranes toward proteins is too high to selectively capture the ovalbumin target [85,86]. Ovalbumin was extracted in aqueous medium from a small sample fragment (<1 mg) after incubation at 10 mM phosphate buffer solution. Ovalbumin reacts with anti-ovalbumin antibody, labeled with glucose oxidase enzyme (Figure 8A). The addition of glucose (as enzyme substrate) and the ferrocenyl redox mediator provides an electrocatalytic oxidation current, with a high signal-to-noise ratio, strictly dependent on the Ovalbumin_OVA concentration levels [18] present in the artwork objects. The electrochemical measurements were performed by applying cyclic voltammetry directly to the original photographic prints (provided by private collections, datable to the nineteenth century), as well as in ancient tempera paintings (datable to fifteenth and eighteenth centuries, provided by restorers in Italy). Results obtained after the addition of glucose demonstrated an electrocatalytic current at the NEEs, incubated with both extracted samples, respectively. The increased current values for the samples were 0.26 μA and 0.27 μA, respectively, and were comparable with those detected on the historical photographic Photo-1 (0.27 μA, without extraction procedures). To validate the results collected with the new immunosensor system, a standard Attenuated Total Reflectance spectroscopy and Fourier Transform Infrared (ATR-FTIR) spectroscopy reference method was applied to Photo-1 and Photo-2 samples, to check the presence of albumen. The ATR-FTIR spectra demonstrated the typical absorption bands characteristic of a protein [18] but the spectroscopic technique is not able to discriminate gelatin and ovalbumin in the same samples, or when in the presence of damaged artwork samples, where the IR adsorption bands may be indistinguishable. During 2013, the same authors detected egg yolk, recording the immunoglobulin IgY on the NEEs, where the sensitive membrane was assembled by a template electroless strategy [87]. Proteins were extracted in aqueous medium, and then IgY selectively reacted with anti-IgY (the latter labeled with horseradish peroxidase enzyme-Anti-IgY-HRP_ Horseradish Peroxidase system), by previous incubation on the NEE surfaces. The binding event of the Anti-IgY-HRP was electrochemically recorded by addition of H_2_O_2_ (HRP enzyme-co-substrate) and methylene blue (enzymatic substrate). A significant increase of the reduction current was obtained, the re-oxidation peak completely disappeared, and the CV profile became a typical sigmoidal curve (which is the full line in Figure 8B (d)). These results clearly underline the presence of IgY, which is the main protein of egg yolk. Considering all data collected by applying both immunosensors, it is reasonable believe that ancient painters used egg white and egg yolk to produce their artworks. In conclusion, it is possible to observe that OVA-immunosensors and IgY-immuno-devices can explore the whole-egg tempera technique but only with a qualitative approach, because neither references [18] nor [87] report on the quantitative analysis using calibration plots/curves, aimed at establishing the concentration levels of proteins present in the original samples. This quantitative analysis is also important in CH, because it helps restorers to select the best restoration and conservation procedures towards artwork objects.

**Figure 9:**
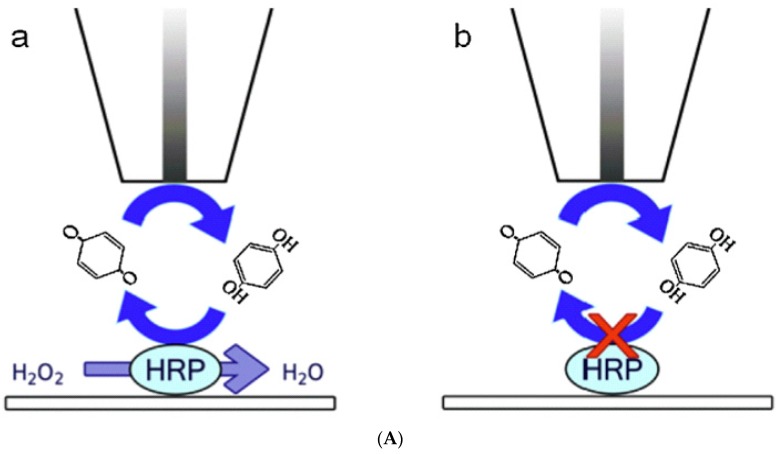

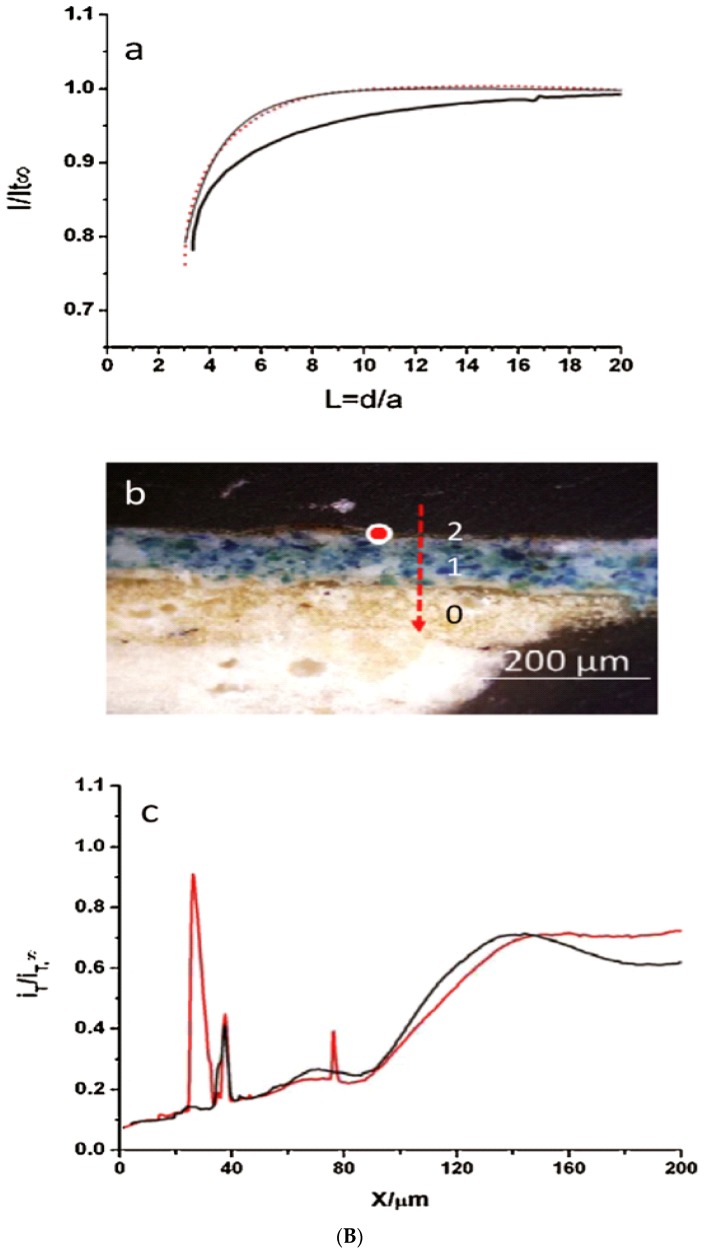
(**A**): is a scheme of the BQ detection. In (**B**), there are: (**a**) SECM approach curve obtained with and without H_2_O_2_; (**b**) represents the optical image of sample; (**c**) represents SECM scan plots, acquired with and without hydrogen peroxide. Reproduced and reprinted with permission from [19].

In this paragraph, the innovative use of SECM for the immunochemical localization of ovalbumin in paint cross-sections is described [19]. The novelty of the proposed analytical method concerns the immunoassay protocol that is directly performed on paint cross-section samples, subsequently positioned into the SECM cell configuration [19], precisely on the tip (i.e., the working electrode, in SECM configuration). The principle of SECM detection and the signal recording is the reduction reaction of benzoquinone (BQ) into hydroquinone (H_2_Q), catalyzed by HRP enzyme. Measurements were performed in feedback mode, because in the presence of hydrogen peroxide (as co-substrate of HRP enzyme), the HRP re-catalyzes the oxidation of H_2_Q to BQ. The BQ concentration levels increase only in the presence of the target protein (ovalbumin that in the past was mixed with yolk or varnishes [88]), detected by SECM through the reduction of the regenerated BQ. The localization of ovalbumin was achieved by the comparison of the measurements recorded without and with the addition of H_2_O_2_ (Figure 9).

The analytical evaluation of the optimization method was undertaken on samples obtained from mock-ups prepared in the laboratory. The optimization protocol provides the detection of the HRP-labeled secondary antibody in the layer (at a distance between 20 and 40 µm from the layer) containing ovalbumin, and the resulting I/A *versus* E/V curves were fitted using the kinetic equation of Corneau–Lefrou [19]. This kinetic equation is highly sensitive and selective towards specific proteins, especially in the presence of ionic elements, as in the case of the colored pigment containing Co^2+^ that did not affect the enzymatic activity of the HRP enzyme. The presence of Fe^3+^ (contained in the hematite pigment) could induce a serious matrix/interference effect, especially after hydrogen peroxide addition, for the typical Fenton reaction. The Corneau–Lefrou fitting curves allows exclusion of the effect of iron as an interfering ion, providing a very selective measure for ovalbumin. The optimized immunosensor was tested on an original sample, collected from a Renaissance painting attributed to Nicolò Baldassarre (ca. 1450–ca. 1510). In this case study, ovalbumin and a varnish layer (the latter related to previous restoration and consolidation treatments) can be selectively recognized thanks to the SECM-based stratigraphy measurements. It is possible to observe a significant current signal variation after the addition of H_2_O_2_ on the external layer (at a distance of between 20 and 40 µm), thanks to the presence of the HRP enzyme and ovalbumin that was unequivocally identified. Larger current values recorded before the addition of H_2_O_2_ are probably related to the interfering presence of varnish. These useful data were requested by conservator scientists and restorers to select the best strategy for the preservation of artwork. SECM results are qualitative and not quantitative, as highlighted by the analysis reported in reference [19], where no calibration plot is reported with a linear range of concentration levels, detectable for ovalbumin, combined with a LOD and sensitivity parameters. Qualitative analysis carried out with immunochemical stratigraphic SECM is excellent for identifying molecular target/probes, but it is not enough for establishing the amount of pigments requiring preservation. Quantitative analysis is an important aspect to consider in CH field applications, because restoration, consolidation, and conservation strategies strictly depend on the precise number of molecular targets, to plan correct experimental remediation protocols on pigment layers. From a qualitative point of view, all the experimental results, collected by immunochemical SECM [19], agree well with those obtained by immunochemical analyses coupled with ChemiLuminescence (CL), SERS-Raman detection [89,90,91], and other immunochemical micro-imaging analyses [92], providing new analytically validated Immuno-SECM-based qualitative methods that are highly specific to the protein qualitative study. At the end of this section, in Table 5, there is a summary of the best analytical performances in terms of qualitative and quantitative analysis and advantages/disadvantages exhibited by all the proposed electrochemical immunosensors.

### 3.3. Portable Electrochemical Biosensors to Apply in CH 

**Figure 10:**
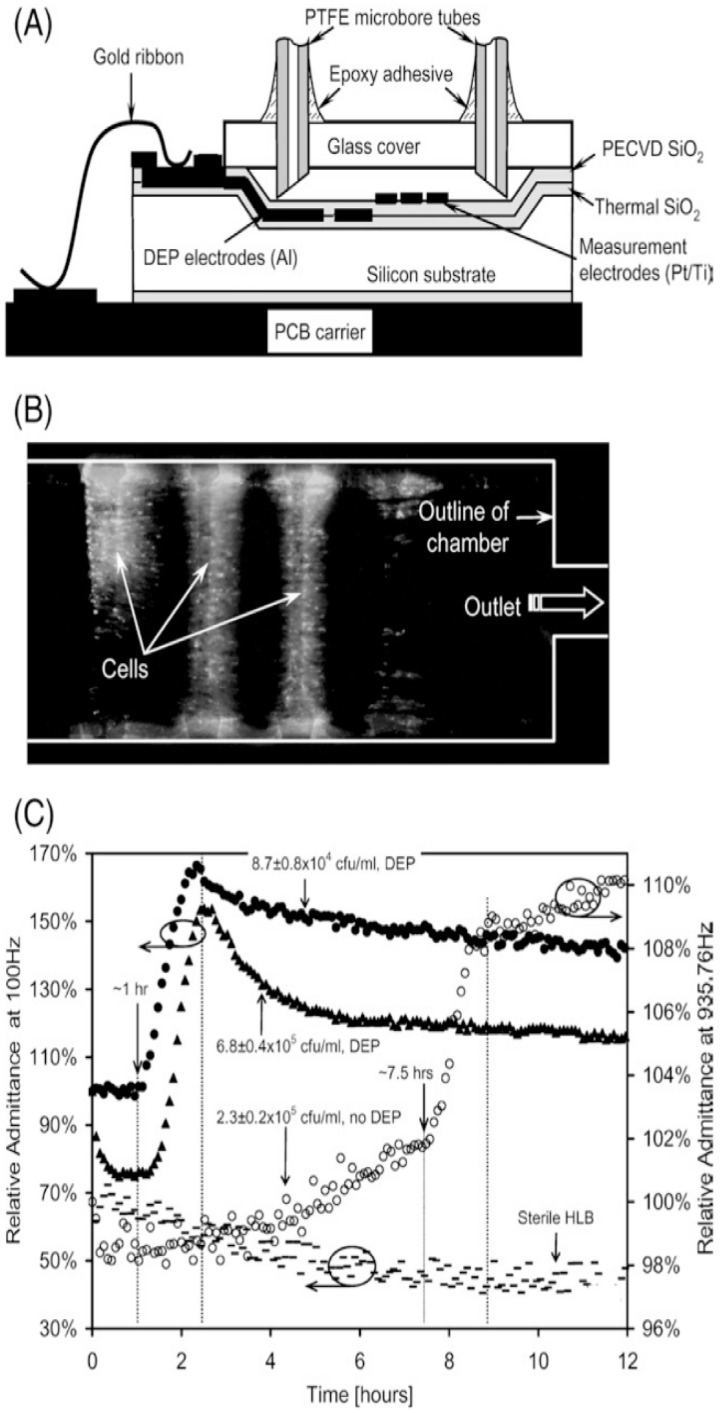
(**A**) Cross-section of the biosensor prototype. (**B**) The analytical signals of fluorescent-labeled Listeria cells. (**C**) Typical impedance growth curves of Listeria cells. Reproduced and reprinted with permission from [21].

In this final section of the review, there are the electrochemical biosensors, with the first prototype represented by impedance biosensor devices. Biosensors are suitable for quantification of pathogenic bacteria [21], mainly responsible for biodeterioration events on CH surfaces (such as biofilms). The assembly of an impedance biosensor tool (namely an EIS device) occurs by immobilizing antibodies, highly specific to the target bacterial cells on the working electrode surfaces. The sensor records the immobilization of bacterial cells by detecting the change in electrochemical properties of the transducers due to the insulating features of the cellular membranes. The EIS signals can be generated and recorded in the presence or absence of redox probes (recording Faradaic and non-Faradaic impedance signals [93]). In 2002, the first impedance biosensor was proposed in the literature [94] that was able to record Faradaic impedance in the presence of an electroactive probe, such as [Fe(CN)_6_]^3−/4−^, with working electrodes (a planar indium-tin oxide-ITO) modified with immobilized anti-*E. coli* antibodies; see Figure 10. An amplification of the electrochemical signal for these biosensor prototypes has been also proposed, applying secondary antibodies conjugated with horseradish peroxide enzyme. This enzymatic system induced a precipitation of insoluble products on the electrode surface (provoking a fouling effect on the working electrode surface), inhibiting electron-transfer reactions and favoring/increasing impedance signals.

The Randles model equivalent electrical circuit (similar to that reported in the first section of this review for the metallic corrosion measurements) can represent and describe the biosensors. The EC shows these electrical/electrochemical elements, such as ohmic resistance (R_s_) of the electrolyte, double-layer capacitance (C_dl_), electron-transfer resistance (R_et_) and Warburg impedance (Z_w_) of the working electrodes. The parallel elements are also located in the EC because they represent the Faradaic process and double-layer charging that also contribute to the electron-transfer processes at the working electrodes. In particular, R_s_ and Z_w_ are bulk properties of the electrolyte and diffusion of the redox target, respectively. C_dl_ and R_et_ represent the dielectric and insulting features at the electrode/electrolyte interfaces, respectively. In particular, R_et_ is the main measured parameter using the impedance biosensors. The immobilization of bacterial cells on the working electrode surfaces could reduce the electron-transfer rates and could increase the electron-transfer resistance (i.e., electrochemical impedance). The total contribution to the electron-transfer resistance (R_et_), after cell attachment on the electrode surfaces, could be due to both contributions of R_e_ and R_cell_. In particular, R_e_ is the electron-transfer resistance of the antibody immobilized on the working electrode, and R_cell_ represents the variable electron-transfer resistance introduced by the attached bacterial cells. Data interpretation returns a typical Nyquist plot, characterized by a semicircle profile, shown on the Z_re_ axis, according to the literature [93]. Extrapolation and elaboration of the semicircle data to lower-frequency values shows an additional bias with the Z_re_ axis that corresponds to the Rs + Ret. The diameter of the Nyquist semicircle represents the electron-transfer resistance, R_et_.

Ruan, et al. [94] showed that this parameter (R_et_) increased with increasing cell concentration, providing great opportunity to carry out the quantitative analysis with a linear range of concentration ranging from 6.0 × 10^4^ to 6.0 × 10^7^ cells/mL and a LOD of 6.0 × 10^2^ cells/mL. An improvement to the quantitative analysis consists of working with label-free electrochemical impedance biosensors, assembled by using the Inter-Digitated Array microelectrode systems (which amplify the output signal, improving the resulting signal-to-noise ratio). Several studies in the literature report on the higher analytical performance of Inter-Digitated Array microelectrodes [95], especially in terms of a wider linear range of concentrations, a lower LOD, and higher sensitivity.

In the following paragraph, there is a description of non-Faradaic impedance biosensor tools, where the impedance measurements were carried out without redox probes (unlike as described above). Radke and Alocilja [96] assembled an impedance biosensor for the detection of *E. coli O157:H7*, applying a high-density microelectrode array with 1700 finger electrodes (to amplify the signal-to-noise ratio). The principle on which the biosensor works is the formation of stable chemical bonds between the bacteria suspension and the immobilized antibodies, increasing the impedance values of the corresponding electrochemical biosensors. This is due to the insulating properties of the adsorbed cell membranes; the latter was recorded over a frequency range of 100 Hz–10 mHz. This means that there is a direct proportionality between the output frequency signals and the adsorbed cells on the biosensor surfaces. This effect (i.e., the direct proportionality between the output frequency signals and the adsorbed cell concentrations) provides a great opportunity to perform a quantitative analysis in the range of 10^4^–10^7^ cfu/mL. Recently, other researchers have significantly improved impedance biosensor tools by applying nanomaterials and new technologies, such as microfluidic tools with inter-digitated microelectrodes. Varshney and Li [97] assembled an inter-digitated impedance biosensor, modified with magnetic nanoparticle–antibody conjugates, for selective detection of *E. coli O157:H7*. The novelty of this prototype lay in the immobilization procedure of antibodies, directly performed on the magnetic nanoparticle surfaces, with the aim to pre-concentrate *E. coli* cells on the bio-transducer surfaces. This pre-concentration strategy allows the lowering of the detection limits for detection of *E. coli O157:H7* in pure culture medium, which resulted in 7.4 × 10^4^ cfu/mL. To improve the detection limit of the biosensor, the same authors [97] assembled this device into a microfluidic chip. The chip contained a small detection chamber with a final volume of 60 nL, and it was also equipped with a gold inter-digitated microelectrode array to amplify the output signals. The microfluidic biosensor also exhibited a low LOD equal to 1.6 × 10^2^ cfu/mL, able to detect bacteria cells when present at low concentration values, corresponding to the biofilm preliminary step formation on CH surfaces and objects.

**Figure 11:**
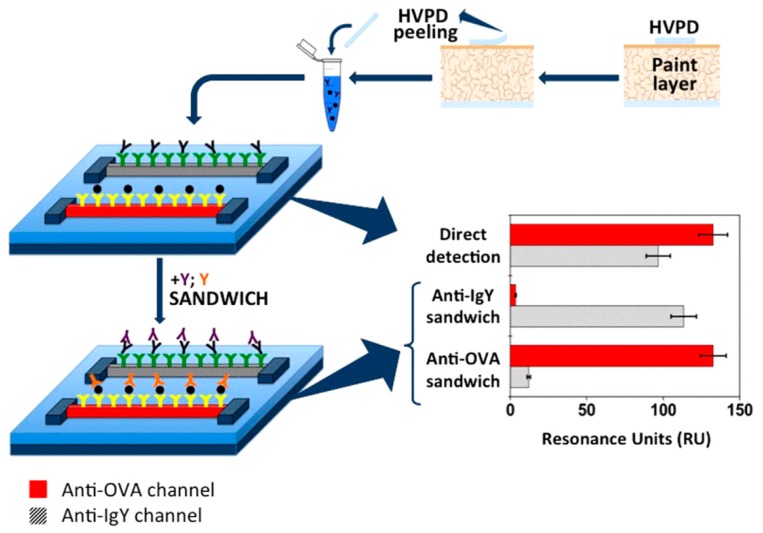
On the left of picture, a typical scheme of the Surface Plasmon Resonance biosensor assembly has been highlighted. On the right, a typical resonance response plot is also shown. Reproduced and reprinted with permission from [22].

Finally, a second electrochemical biosensor prototype, which will conclude this review, consists of a Surface Plasmon Resonance-based biosensor (Figure 11), for the first time applied in CH conservation science, to recognize albumen, yolk, or their mixtures in HVPD extracts [98]. HVPDs have been recently developed and successfully applied for the selective removal of environmental patinas that affect the paintings on art objects and this HVPD contained hydrolyzed polyvinyl acetate, borax, and water [98]. The assembly of a Biacore X™, carboxymethylated dextran CM5 biochips, gives an opportunity to perform Surface Plasmon Resonance measurements, immobilizing antibodies on CM5 biochips by amino-coupling reaction, according to the literature [98]. This proposed biosensor can be also regenerated (providing a new concept for fast cleaning/sampling methods for CH conservation), by using NaOH aqueous solution at a concentration of 100 mM, for 30 s.

**Figure 12:**
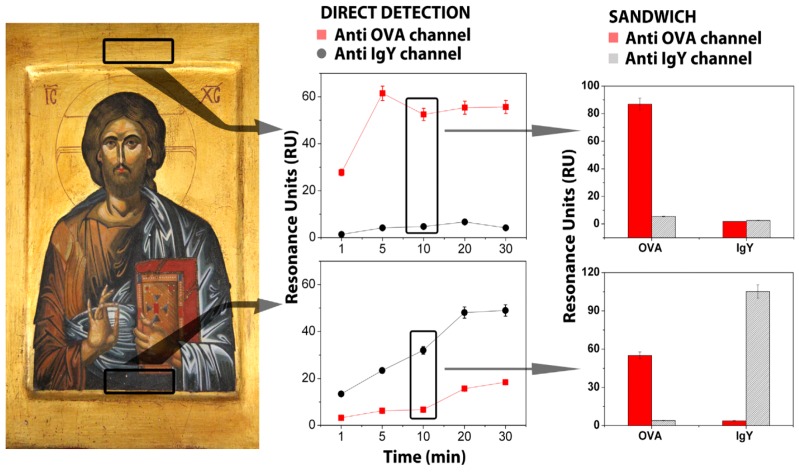
Original sample to test biosensor (left). In the center, SPR-biosensor data relative to Highly Viscous Polymeric Dispersion extracts from the gilded and painted zone. The graphs to the right show the processed data. Reproduced and reprinted with permission from [22].

After measurements, the collected results showed a selective response toward albumen-relative (anti-OVA) receptor (equal to 89.13.9 RU), providing nonspecific signals on the (anti-IgY), with a specific: nonspecific ratio of 36.33. When in the presence of a pure and fresh yolk sample, specific (anti-IgY) output signal was recorded around 36.61.5 RU, while the unspecific signal was observed only for (anti-OVA) channel equal to 35.71.4 RU. Moreover, the output signal for yolk detected on (anti-IgY) channel was very low, and to improve it, a sandwich strategy was successfully applied in the presence of pure matrices (albumen or yolk) or mixtures of them (albumen/yolk present in the same samples). The direct assay of albumen/yolk mixture recognized the presence of both chemical analytes (140.0 ± 5.6 RU for anti-OVA channel, 123.3 ± 4.9 RU for anti-IgY channel), with higher signal-to-noise ratio intensities, when compared with those obtained in the presence of pure matrices (only one element per sample). This specific effect is probably a synergistic extraction event that occurred between albumen and the HVPD-extracting medium. Biosensor results are highly selective towards organic components contained in artwork pigments and, for this purpose, the test was conducted on a modern “sacred icon”, characterized by the presence of gilded and painted areas, naturally aged for 12 years (Figure 12), and on an eighteenth-century Italian painting sample.

On a modern “sacred icon” original CH sample, five equivalent aliquots of HVPD (50 mg for each aliquot) were applied to both areas, as gilded and painted zones, for a contact time ranging from 1 to 30 min. The preliminary experiments, carried out to standardize the correlation among the contact time of HVPD and the artwork surfaces, the extracted molecules, and the Surface Plasmon Resonance biosensors, highlight the presence of albumen on gilding zones, and the presence of mixture (albumen and yolk) on the painted areas. Experimental data/results collected on the modern sacred icon surfaces demonstrated that after only 5 min (as contact time between the extracted organic samples and the biosensor transducers) it is possible to collect all the total amount of ovalbumin, contained in the painting samples. The experimental results confirmed that very short application times were enough to extract useful information, and, at the same time, they were ideal for avoiding invasive treatments on original artwork surfaces. Given this aspect, the authors [22] also applied the sandwich test for 10 min, confirming the presence of the albumen on the gilding area of the modern sacred icon surface, whereas the painted area contained yolk–albumen mixture, respectively. To complete this study, the same authors [22] tested the new device on an eighteenth-century Italian painting sample, of which the construction technique was completely unknown. The negative control sample was a standard painted surface without egg proteins. The latter was used for comparative study. The experimental results successfully confirmed the presence of egg compound in the paint layer, unequivocally identified as pure egg yolk. The Surface Plasmon Resonance biosensor detected a direct output signal of yolk with a nonspecific signal on the anti-OVA channel, which was very well resolved by the sandwich assays. The results on the eighteenth-century Italian painting sample was also confirmed by the absence of Surface Plasmon Resonance signal, when in the presence of the negative control (i.e., a standard painted surface without egg proteins), provided useful information about the use of yolk-based tempera in the case of eighteenth-century Italian paintings. A summary of the best analytical performance is in Table 6, for all the electrochemical biosensors reported here.

## 4. Conclusions

In this review, two main aspects of electrochemical techniques/tools applied for CH analysis have been considered. The first one concerns EIS for the evaluation of metal corrosion events (belonging to CH, such as outdoor layers or indoor coating on artwork surfaces). This represents an important field to explore with EIS methodology because metallic artwork surfaces must be protected from atmospheric damages and deterioration events, especially in terms of oxidation processes. EIS provides qualitative analysis/identification of metals contained in CH layers, and simultaneously offers important information on the redox status of metallic coating, which is strictly related to their conservation conditions over time. No other analytical techniques devoted to the analysis of metals, such as Atomic Absorption Spectroscopy (AAS) or Inductively Coupled Plasma-Mass Spectrometry (ICP-MS), are able to provide information on the redox status of metals in CH objects. The redox features of the metals in CH field applications represent an important aspect to communicate to restorers and conservation scientists, because they provide the right knowledge and awareness for choosing the best restoration and conservation strategy for artwork objects under investigation. If these aspects represent a strong point for EIS, the miniaturization of equipment and the processing of output signals/elaboration data constitute two non-advantageous aspects that do not make this technique routinely applicable in the field of CH. This review shows that the number of scientific publications is high and therefore soon the EIS technique will become fundamental in the field of CH conservation and preservation. A first example is reported in this review through solid electrolytes inside cells with more flexible geometries, which are more adaptable for the investigation of historically interesting surfaces. The second aspect is an overview of the electrochemical small portable devices for in situ analysis, especially required in the presence of unmovable CH objects and surfaces. In this section, electrochemical sensors, immunosensors, and biosensors have been reported and described in detail. Among them, electrochemical sensors (based on GO/SPEs) and electrochemical impedance-based biosensors exhibit the best analytical performance, especially under a qualitative and quantitative point of view. In particular, GO/SPEs provide a great opportunity to selectively quantify As(III), total As elements, Fe(III), and total Fe elements contained in organic colored pigments and/or earth, present in ancient deteriorated leather samples, with the same sensitivity and LOD exhibited by a reference-quantitative analytical technique, such as ICP-MS. The EIS-based biosensors provide the possibility of determining proteins, organic components, and egg yolk with low detection limit, high sensitivity, and fast response time (minimizing the electrochemical interferences).

To conclude, EISs represent smart portable tools, but only for qualitative screening analysis. This is not at all a negative aspect; on the contrary, it offers an analytical strategy of preliminary screening that allows the obtaining of qualitative information that is useful to reduce the amount of analysis (in some cases also the number of samples), thus allowing measurements to be taken in real time, at low cost. The great advantage of the innovative imaging Immunosensing techniques is the stratigraphic analysis of the layer surface, to precisely allow the localization of colored pigments and organic components.

## Figures and Tables

**Table 1:** The most representative cases of studies investigated by ECs for a correct EIS profiles/data evaluation.

Original Metal Samples	Equivalent Circuit (ECs) and Electrical Elements	Real Case of Study: Original Metal Coatings/Layers	References
*Clean metals*	Randles circuit with R_e_ that is the electrolyte resistance; CPE_dl_ is the double-layer capacitance; R_ct_ is the charge transfer resistance, and finally W is the Warburg impedance for copper ions, during their diffusion through the oxide film	The Randles circuit was analytically standardize and validated, by using the standard corrosion events of copper in slightly mineralized neutral aqueous solutions (such as tap water, as conventional working medium)	[60,61]
*Clean metals*	A variation of the previous Randles circuit consists of the charge transfer resistances of cathodic and anodic processes, are in parallel in different branches of the circuit	The EIS spectra, acquired with the modified Randles circuit, was applied to recorded EIS profiles for artistic bronze coupons in artificial rain	[60]
*Clean metals*	Other authors have applied a simple two nested (R-CPE) couple circuit, but fitting results showing the exponent of the second CPE close to 0.5, suggesting a diffusion impedance, both in copper and brass	Copper and brass surfaces for application of the two nested (R-CPE) couple of electrochemical circuits	[62,63,64]
*Metals with patinaCopper and alloys*	The first EC (where the pair CPE_dl_-R_ct_ is not considered to be electrical equivalent element) represents the double-layer patina morphology, quite similar to the anodized layers in aluminum model, which consist of a thin barrier layer covered by a porous outer layer	This equivalent circuit describes the outdoor copper and bronze patinas, exhibiting a double-layer structure. In particular, the inner layer contains cuprous oxide materials and an outer layer, appears more porous for the presence of different cupric compounds, depending on the environment to which the object is exposed and located	[65]
*Metals with patina Copper and alloys*	The second EC with two nested (R-CPE) couple circuit, represents the impedance of the inner and outer patina layer. Considering an exponent value of 0.5 for the CPE in the inner layer, a Warburg/W impedance, could replace the CPE final circuit.	The same EC was applied to study the response of bronze roman coins and natural copper patinas, formed during 1–3 years in Chile in different environments, with different thickness and porosity depending on their location	[66]
*Metals with patina Copper and alloys*	Three nested (R-CPE) circuits were reported in the literature, to explain the electrochemical output signals regarding artificial patinas, putting in Na_2_SO_4_-NaHCO_3_, as working electrolytes. The first (R-CPE) pair represents the resistance and capacitance of the patina, the second (R-CPE), at intermediate frequencies, represents the corrosion process on the metal surface, while third (R-CPE) couple that corresponds to the low-frequency loop is explained as a result of oxidation–reduction processes of the corrosion products taking place at the electrode surface	A first example of this three nested (R-CPE) circuits is the EIS spectrum profiles of samples, collected by a brass object, excavated from the archaeological area of Tharros, in 0.1 M NaCl (as working electrolyte). Another example concerns archaeological bronze coins, working with NaCl 0.3 M-5% agar electrolyte and mineral water. This EC is depicted in a different order (R_e_[(R_ct_-CPE_dl_)(R_p_l-CPE_pl_)]) that is mathematically. Equivalent as EC and which best represents the original sample	[67,68,69,70,71]
*Metals with patina Iron and steel*	Two-cell EC have been applied to describe the impedance of the two interfaces: metal/rust layer and rust layer/electrolyte, respectively. A third time constant seems to be present at low frequencies, applying the R(RC(C[RW])) model and performing measurements in the G-PE cell	Regarding the Two-cell EC circuits electrochemical studies were carried out on weathering steel sculptures from Adriana Veyrat; Politecnico di Torino performed EIS measurements on historic iron surface/coating belonging to the Notre-Dame Cathedral of Amiens and the Metz Cathedral, in France	[72]
*Evaluation of coatings*	A metal-coating system is a capacitor and a resistance in parallel, according to the capacitance (C_coat_) and resistance (R_coat_) of the metal coating in series with the resistance of the working electrolyte, (R_e_). In highly protective coatings, R_coat_ is very high and the system becomes C_coat_ in series with R_e_ (no current crosses the resistance). When the coating deteriorates the circuit, changes and the main electric components/elements are C_dl_ (the double-layer capacitance and R_ct_ (the charge transfer resistance) of the corrosion process that occurs at the metal-electrolyte interface. This circuit was applied to the characterization of organic coatings, including varnishes and waxes for bronze and historic steel artwork objects	Not reported cases of studies on original samples	[73,74,75]
*Evaluation of inhibitors*	The EC circuits are the same of the clean surfaces, only differing in the values of different parameters	Not reported cases of studies on original samples	[76,77]

**Table 2:** Main characteristics and analytical performance of Setup 1 and Setup 2 proposed in the first section of the review.

Electrochemical Configurations	Quantitative Analysis	Qualitative Analysis	Main Advantages	Main Disadvantages
*Setup1*:	----------------------------	✓	easy sensor geometry for in situ manipulation	Stiff (not flexible) contact probe and the liquid conductive electrolytes provoke electrochemical contact problems toward the surface of cultural heritage, compromising the final electrochemical measurements
*Setup2*:	----------------------------	✓	Flexible cell geometries suitable for CH surfaces (with significant roughness) Solid conductive electrolytes to guarantee the electrochemical/electrical contact toward CH surfaces	The difficulty of processing the acquired experimental data by ECs, working with Nyquist plot

✓ Qualitative analysis is present in each electrochemical small-sensor prototype; --------------: this symbol means that the specific parameter is not reported in the paper.

**Table 3:** Comparative study between GO/SPE and ICP-MS for As(III) and Fe(III) quantification.

Sample As(III)	GO/SPEs by SWCSV (μg/L)	Total Inorganic As: [As(III) + As(V)] Total Inorganic Fe: [FeO + Fe_3_O_4_ + Fe_2_O_3_] (μg/L)	ICP-MS (μg/L)	P Value (t-Test)^a^
1	992 ± 2	101 ± 2	100.80 ± 0.5	0.01
2	100 ± 1	100 ± 1	100.00 ± 0.02	0.03
3	102 ± 2	103 ± 1	103.05 ± 0.5	0.02
Sample Fe(III)				
1	399 ± 1	400 ± 1	400 ± 1	0.04
2	400 ± 2	400 ± 2	400 ± 0,2	0.01
3	402 ± 1	403 ± 0.3	403 ± 1	0.03

^a:^ two-sided t-test to compare means from heteroscedastic data. Data reported on Table 3 are reproduced and reprinted with permission from [16].

**Table 4:** Main characteristics of the most important electrochemical sensors applied in CH, mainly presented in this review.

Electrochemical Devices	Quantitative Analysis	Qualitative Analysis	Main Advantages	Main Disadvantages
Raman spectrometer, equipped with a potentiostatic apparatus	LOD = 5 ppm (mg/L) for melamine	✓	low cost measurement, no-time consuming analysis, high sensitivity, low detection of limit	Non-electroactive pigments and organic binders cannot quantify by electrochemistry
Electrochemical cell, which also works like a Bragg tool	----------------------------	✓	to detect different crystallographic phases during serious corrosion events, to detect the corrosion potential E_corr_, both parameters, unequivocally provide important information on the nature of metallic corrosion phenomena	not all the corrosion products, to which different crystallographic phases belong, have electrochemical activity, therefore the disadvantage is that there is only qualitative crystallographic information
Screen-Printed Electrodes (SPEs) chemically modified with Graphene Oxide (GO); GO/SPE	See Table 3	✓	Voltammetric techniques are useful to understand two important aspects, as -the chemical-physical composition of the colors; -the oxidation status and conservation conditions of metallic elements contained in organic pigments, useful to select the best restoration strategies	Particularly useful only in the case of metals contained in inks, colored pigments and organic compounds The absence of metals makes the application of this device more difficult, as the organic component (very often) does not have a marked electrocatalytic activity
micro-sample coatings in Paraloid B72 film-modified electrodes, combined with	----------------------------	✓	micro-sample coatings in Paraloid B72-film provide the possibility of pre-concentrating the pigments to increase the Signal/Noise ratio, especially in the presence of traces of organic materials	micro-sample coatings in Paraloid B72-film are not so selective and for this purpose several interferences and/or passivation/fouling effects can occur during analysis/measurements

✓ Qualitative analysis is performed; --------------: this symbol means that the specific parameter is not reported in the paper.

**Table 5:** Summary of the best analytical performance for the immunosensors described and compared in this section of the review.

Immunosensor Prototype	Quantitative Analysis	Qualitative Analysis	Main Advantages	Main Disadvantages
Nano-Electrode Ensembles (NEEs) immunosensors for the detection of ovalbumin in paintings	✓	-----------------------------------	High selectivity and specificity for the proteins and organic binder recognition, more than FTIR traditional method	No quantification of the organic component in paintings and this could represent a lack of useful information for restorers
Nano-Electrode Ensembles (NEEs) immunosensors for the detection of egg yolk in tempera and paintings	✓	-----------------------------------	High selectivity and specificity for the proteins and egg yolk recognition, more than FTIR traditional method	No quantification of the organic component in paintings and this could represent a lack of useful information for restorers
Scanning Electro-Chemical Microscopy (SECM) immunosensors	✓	------------------------------------	Immunochemical stratigraphic SECM is excellent to selectively identify the organic components in the painting layers	Low reproducibility due to the passivation/fouling of the scanning/electrode-based tip Low Signal/to Noise Ratio

✓ Qualitative analysis is performed; --------------: this symbol means that the specific parameter is not reported in the paper.

**Table 6:** Summary of the best analytical performances for the electrochemical biosensors.

Biosensor Prototype	Quantitative Analysis	Qualitative Analysis	Main Advantages	Main Disadvantages
Impedance biosensor devices, recording Faradaic signal, in the presence of the electroactive probes	✓	Linear range of concentration: 6.0 × 10^4^–6.0 × 10^7^ cells/mL; Limit Of Detection (L. O. D.): 6.0 × 10^2^ cells/mL.	High selectivity and sensitivity toward the organic binder recognition, more than FTIR traditional method. The biosensor regeneration opportunity	The inadequacy, in some case studies, of the Randles model equivalent electrical circuit to describe the electrochemical impedance biosensor prototypes
Impedance biosensor devices, recording non-Faradaic signal, without the electroactive probes	✓	Linear range of concentration: 10^4^–10^7^ cfu/mL; Limit Of Detection (L. O. D.): 1.0 × 10^2^ cells/mL.	High Signal/Noise ratio Lower LOD	Possible Fouling and passivation phenomenon with interference on the output signals due to the stable chemical bonds between bacteria suspension and the immobilized antibodies
Surface Plasmon Resonance-based biosensors	✓	The direct assay of albumen/yolk mixture recognized the presence of both chemical analytes (140.0 ± 5.6 RU) for (anti-OVA) channel, (123.3 ± 4.9 RU) for (anti-IgY) channel.	The experimental results confirmed that very short application times were enough to extract useful information and, at the same time, they were ideal for avoiding invasive treatments on original artwork surfaces	The main analytical problem could be related to the interference when in absence of Highly Viscous Polymeric Dispersions (HVPDs) extracts from original samples

✓ Qualitative analysis is present in each electrochemical small-sensor prototype.
