# Correction: Smart Electrochemical Portable Tools for Cultural Heritage Analysis: A Review. *Sensors*, 2019, *19*, 4303

**DOI:** 10.3390/s20010159

**Published:** 2019-12-25

**Authors:** Federica Valentini

**Affiliations:** Sciences and Chemical Technologies Department, Tor Vergata University, via della Ricerca Scientifica 1, 00133 Rome, Italy; federica.valentini@uniroma2.it

The authors wish to make the following corrections to this paper [1]:

1. The graphical abstract should be replaced with


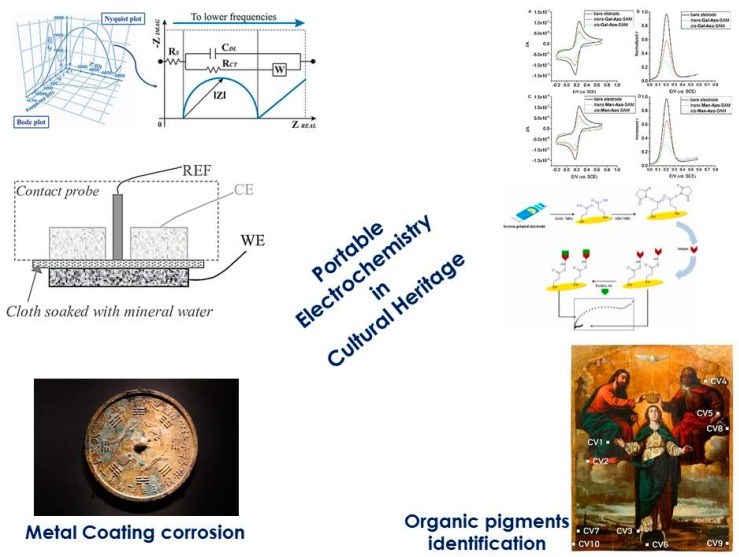


instead of


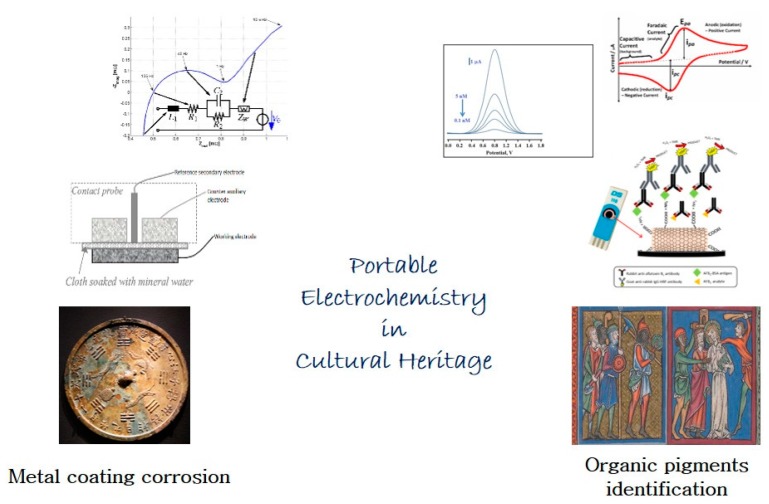


2. The citation in Figure 2 is incorrect and should be revised from Reference [30] to [28]. The correct caption should be “**Figure 2.** A contact probe (CP), reproduced and reprinted with permission from [28].” Furthermore, Reference [28] should be revised to “Letardi, P.; Albini, M.; Joseph, E. EIS measurements for treatment testing: the case of a bio-based method applied on outdoor bronze statues in Switzerland. Available online: https://www.scienceopen.com/hosted-document?doi=10.14293/S2199-1006.1.SOR-.PPANDZU.v1 (accessed on 27 September 2019).”

3. The third paragraph in Section 2 should be revised to read “Setup 2 is a gel cell, with a silver/silver chloride secondary electrode and agar or agarose as the gelling agent [30–32], which is useful for immobilizing alkaline chloride solutions, thus simulating what happens inside the pores of concrete and plaster surfaces [33]” from “Setup 2 is a gel cell, with silver/silver chloride secondary electrode and agar or agarose as gelling agent [31,32] useful for immobilizing alkaline chloride solutions, simulating what happens inside the pores of concrete and plaster surfaces [33].”

4. The citation in Figure 3 is incorrect and should be revised from Reference [30] to [50]. The correct caption should be “**Figure 3.** (**A**) Solid agarose gel electrolyte: scheme (**up**) and photograph (**down**); (**B**) zoom of a solid agarose gel. Reproduced and reprinted here with permission from [31] and [50], respectively.”

5. The citation in Figure 4 is incorrect and should be revised from Reference [41] to [30]. The correct caption should be “**Figure 4**. (**a**) EC showing an ideal metal-coating system; (**b**) a damaged coating; and (**c**) different EC schemes proposed to represent archaeological copper alloys. Reproduced and reprinted with permission from [30].”

6. Citations should be added in Scheme 2. The correct caption should be “**Scheme 2.** Equivalent Circuits (ECs) reported in Table 1, in particular: (**A**) represents the AC: Alternative Current Impedance Spectroscopy for copper in the simulated tap water. (**B**) Represents the electrical equivalent circuit used for fitting the EIS data in the presence of a copper/alloy-coated patina. (**C**) Shows the EIS measuring principle and the corresponding EC carried out on the iron/steel-coated patina. (**D1**) Cell design scheme (**left**); bronze coupons used for the electrochemical tests (**middle D2**) and the equivalent electrical circuits (**D3**) were used to analyze the EIS data. (**a**) is the equivalent circuit with two nested CPE-R pairs, and (**b**) is the second CPE replaced by a generalized finite-length Warburg impedance. Reproduced and reprinted with permission from [32,60,67,72], respectively.”

7. Figure 10 should be replaced with

**Figure 10:**
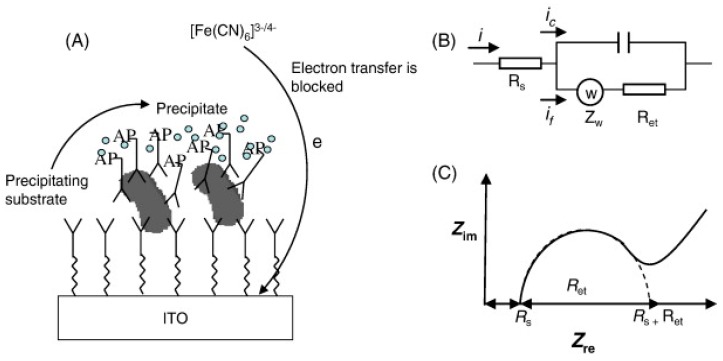
(**A**) The operating principle concerning the electrochemical impedance biosensor for bacteria quantification. (**B**) The Randles model equivalent circuit, and (**C**) the typical Nyquist plot (Z_im_ vs. Z_re_) of the Faradaic impedance spectrum of the electrochemical device when in the presence of the redox probe. Reproduced and reprinted with permission from [94].

instead of

**Figure 10:**
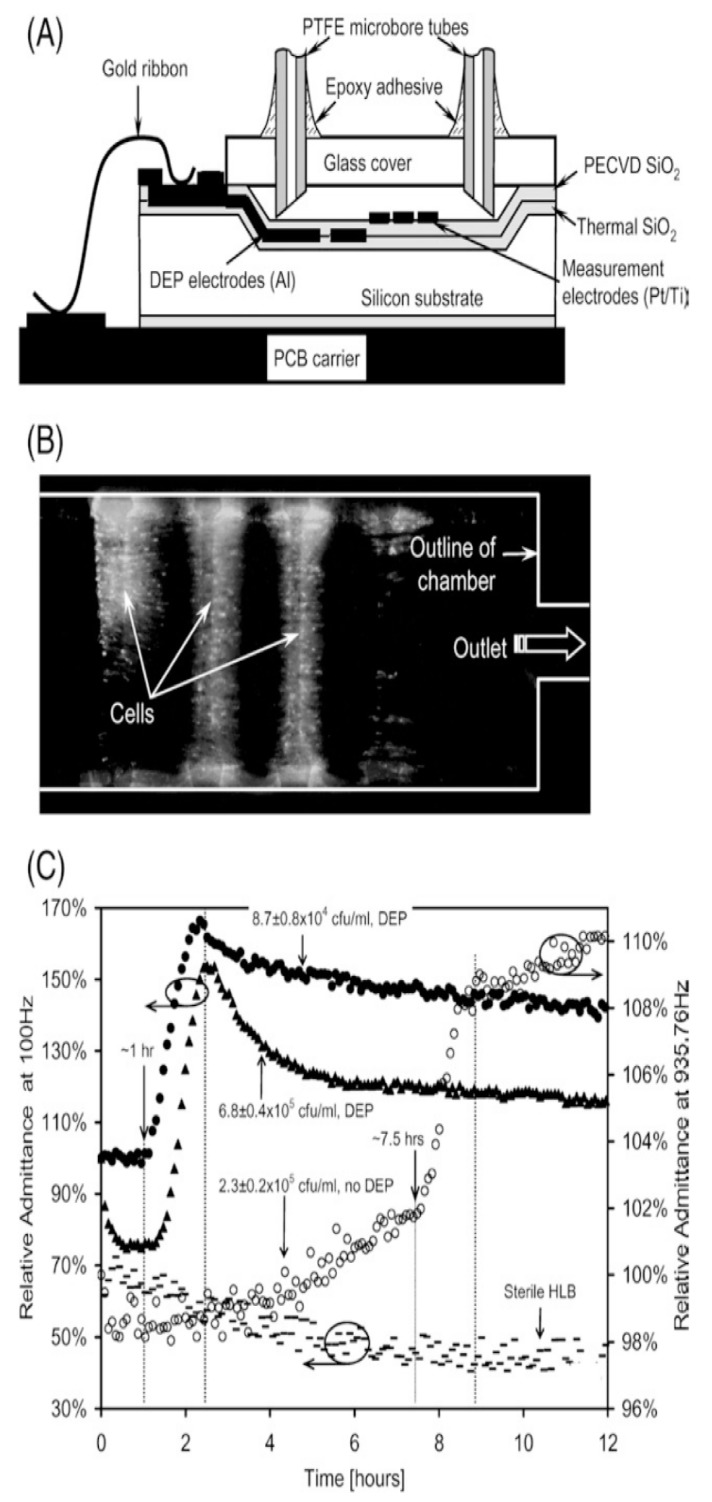
(**A**) Cross-section of the biosensor prototype. (**B**) The analytical signals of fluorescent-labeled Listeria cells. (**C**) Typical impedance growth curves of Listeria cells. Reproduced and reprinted with permission from [21].

The authors would like to apologize for any inconvenience caused to the readers by these changes.
